# ﻿Two new species of *Bryocamptus* (Copepoda, Harpacticoida, Canthocamptidae) from the Russian Arctic and comparison with *Bryocamptusminutus* (Claus, 1863)

**DOI:** 10.3897/zookeys.1138.90580

**Published:** 2023-01-05

**Authors:** Aleksandr Novikov, Dayana Sharafutdinova, Elena Chertoprud

**Affiliations:** 1 Kazan Federal University, Kremlyovskaya St. 18, 420008 Kazan, Russia Kazan Federal University Kazan Russia; 2 Department of Hydrobiology, Biological Faculty, M.V. Lomonosov Moscow State University, Leninskie Gory, Moscow 119991, Russia M.V. Lomonosov Moscow State University Moscow Russia; 3 A.N. Severtsov Institute of Ecology and Evolution, Leninsky Prospect, 33, Moscow 119071, Russia A.N. Severtsov Institute of Ecology and Evolution Moscow Russia

**Keywords:** Arctic invertebrates, biodiversity, intraspecific differences, sensillae and pores, sexual arms race

## Abstract

Two new species of *Bryocamptus* Chappuis, 1929 from the Russian Arctic from the *Bryocamptusminutus* species group are described: *Bryocamptusputoranus***sp. nov.** and *Bryocamptusabramovae***sp. nov.** A complete morphological comparison of the new species with the type species *Bryocamptusminutus* (Claus, 1863) was carried out. Significant interspecific differences were shown at the level of microcharacters, such as integumental sensillae and pores, ornamentation of segments of mouthparts and swimming legs, and pores on swimming legs. A significant correlation has also been shown in the shape of the caudal rami of the females and the antennules of the males, which is likely caused by an evolutionary sexual arms race. *Bryocamptusputoranus***sp. nov.** and *B.minutus* have a similar structure of caudal rami, but completely different male antennules, which may indicate a convergent origin of modifications and highlights the importance of depicting male antennules in the species descriptions.

## ﻿Introduction

Recent studies have shown a very low level of knowledge of the freshwater Harpacticoida fauna in the Russian Arctic. Previously, we discovered several new species from the genera *Moraria*, *Bryocamptus*, *Maraenobiotus*, *Canthocamptus* (Novikov and Sharafutdinova 2020; Novikov et al. 2021). In this paper, we consider three species of *Bryocamptus* from the Bryocamptus (Bryocamptus) minutus (Claus, 1863) species group with descriptions of two new species from the Arctic. This group was originally identified by K. Lang on the basis of a one-segmented mandibular palp (1948). Unfortunately, most descriptions of the freshwater Canthocamptidae of the last century were very often incomplete or quite poor. Often, figures and descriptions of mandibles were not given at all. Therefore, at this stage, it is impossible to clearly determine which species are included in this group, or to conduct a fully-fledged taxonomic analysis of the group, and even more so of the genus.

In modern taxonomy, in addition to molecular genetic analysis, an important component is the study of microcharacters that were generally not taken into account earlier. In recent years, more and more data were collected on the wide distribution of complexes of cryptic and pseudo-cryptic species of copepods (Lajus et al. 2015; Kochanova et al. 2021). Microcharacters make it possible to distinguish, more or less reliably, between such species (for example Hołyńska and Dahms 2004; Stoch and Bruno 2011; Karanovic and Krajicek 2012; Karanovic and Lee 2012). Such characters include ornamentation of limb segments, the structure of the somite integument, and in particular, sensillae and pores. In this work, we tried to present the most detailed description of three closely related species from different parts of the Palearctic. Despite the obvious and well-observed differences, we focus on small characters for the purpose of their possible future use.

## ﻿Materials and methods

Material from the Lena River Delta (north-eastern Siberia) was collected during the “Lena-2019” expedition. Crustaceans from the Putorana Plateau were collected in August 2021 during an expedition by Moscow State University in the Natural Reserve Putoransky. In the first case a small plankton net (mesh size 80 μm) was used for collection. In the second case samples were taken with small plastic tubes (radius 1.2 cm). A description of the collection of materials in Estonia is given in the work of Fefilova (2010).

Samples were fixed in 4% formalin or 96% ethanol. Specimens were dissected under a stereomicroscope, with each element being placed in glycerol under a separate coverslip. Pieces of plasticine are used on the underside of the coverslip to prevent damage to the element. Next, series of photographs were taken using a USB camera, which were merged in the Helicon Focus 6 program. The drawings and photographs were taken with a microscope (LOMO Micmed 2, Russia). Rough drawings were obtained from printed photographs of elements, and the final drawings were prepared using the free program Inkscape 1.0.

All depicted limbs and other elements were examined from at least three individuals of each species: two females and one male, with the exception of the labrum and paragnaths, which were studied from only one individual. The numbering of pores and sensillae on somites is original and based on the structure of the integument of several freshwater species of Canthocamptidae. Roman numerals (for pores) or Arabic numerals (for sensillae) are used for numbering integumental elements. The designations for cephalothorax sensillae C, P, and L are used to simplify homology. Group P is the sensillae adjacent to the edge of the cephalothorax. Group C is the sensillae, which are located near the medial axis and the dorsal window. The notation L is used for all other sensillae.

Nomenclature and descriptive terminology follow Huys and Boxshall (1991), terminology of genital fields follows Moura and Pottek (1998), terminology of mandibular structure follows Mielke (1984), terminology and homology of maxillary structures follow Ferrari and Ivanenko (2008). The armature formulae of swimming legs are given according to Lang (1934). By the term “helle Stelle” we mean the inner cuticular disc at the base of the apical caudal setae (sensu Lang 1948).

For *B.abramovae* sp. nov. and *B.putoranus* sp. nov. only features that differ from *B.minutus* are described. All material was deposited in the Zoological Museum of Kazan Federal University (**KFU**).

### ﻿Abbreviations used in the text

**A1** antennule

**A2** antenna

**Ae** aesthetasc

**Acr** acrothek

**Ap** apophysis

**P1–P6** legs 1–6

**PS2–PS5** pedigerous somites 2–5

**Exp1–Exp3** first–third segments of exopod

**Enp1–Enp3** first–third segments of endopod

## ﻿Taxonomic account

**Subclass Copepoda H. Milne Edwards, 1840**

**Order Harpacticoida Sars, 1903**

**Family Canthocamptidae Sars, 1906**

### ﻿Genus *Bryocamptus* Chappuis, 1929

#### ﻿ Subgenus Bryocamptus

Taxon classificationAnimaliaHarpacticoidaCanthocamptidae

Chappuis, 1929

ADC42B81-DD26-52E3-8A4A-B967ABA4BA5D

##### Remarks.

Bryocamptus is a very large genus with ~ 135 species and subspecies in four subgenera: B. (Arcticocamptus) Chappuis, 1929, *B.* (*Bryocamptus)* Chappuis, 1928, B. (Echinocamptus) Chappuis, 1929 and B. (Rheocamptus) Borutzky, 1952. Additionally, two subgenera were earlier designated as not valid B. (Limocamptus) Chappuis, 1929 and B. (Pentacamptus) Wiley, 1934.

In our opinion, this is one of the genera of the family most in need of revision. The first reason is that there are no clear diagnostic characters for the entire genus. Previously, this character was the two-segment exopod A2; however, this character is plesiomorphic for the entire family Canthocamptidae, so it may be an adequate solution to separate at least part of the subgenera into separate genera. The second reason is the blurred line between B. (Bryocamptus) and B. (Rheocamptus). Borutzky (1952) in the differences between these subgenera indicates the difference in segmentation of the endopods P1–P4, which again contrasts plesiomorphic and apomorphic characters. In our opinion, an essential part of the B. (Rheocamptus) species should in fact be transferred to the type subgenus.

Unfortunately, at the moment we do not have enough data and material to revise the subgenera, so in this work we adhere to the classification given by Dussart and Defaye (1990).

#### ﻿ Bryocamptus (Bryocamptus) minutus

Taxon classificationAnimaliaHarpacticoidaCanthocamptidae

(Claus, 1863)

721DB59E-6F34-503B-B170-20E96BFFC3FE

##### Subspecies.

B. (B.) minutus minutus (Claus, 1863), B. (B.) minutus schizodon (Mrázek, 1893).

##### Nomen dubium.

B. (B.) minnesotensis (Herrick, 1884).

##### Remarks.

Bryocamptus (B.) minutus is a taxonomically rather complex species due to a rather long history of study and wide distribution. According to Article 45.6 of the International Code of Zoological Nomenclature, a number of forms of this species must be treated as separate subspecies (ICZN 1999). However, in the case of B. (B.) minutus vejdovskyiformis Thallwitz, 1916, this is probably a form that does not have subspecies status and is either an aberrant specimen(s) or simply variability (Thallwitz 1916). Simple dentiform and bifid spinules are also found in other related species, both within the same population and in one individual. This has been described in *B.hutchinsoni* Kiefer, 1929 (Wilson 1956), *B.vejdovskyi* (Mrázek, 1893) (Reed 1990) and also in *B.putoranus* sp. nov. (in this article).

A number of authors noted variability in the number of outer spines on the third exopodal segment of P4, which was the reason for Lang’s description of the forms: B.minutusf.typica Lang, 1957 and B.minutusf.bispinosa Lang, 1957 (Lang 1957). We suggest that these forms do not have a taxonomic rank, since such variability is common for this group of species.

Another form of B.minutusf.simplicidentata (Willey, 1934) has been synonymized with *B.hutchinsoni* based on structure of caudal rami (Wilson 1956) but although figuring mistakenly and without literature support as valid in WORMS database (Walter and Boxshall 2021).

A rather interesting finding is described from the Iberian Peninsula as *B.minutus* (Caramujo and Boavida 2009). Based on the depicted limbs, it can be assumed that this is either *B.minutusschizodon* or a separate species. It differs from *B.minutusminutus* in the two-segmented endopod P2, short bifid spinules on the anal operculum, and slight displacement of the caudal setae to the ventral side of caudal rami. In general, these characters are already enough to distinguish a separate species.

#### ﻿ Bryocamptus (Bryocamptus) minutusminutus

Taxon classificationAnimaliaHarpacticoidaCanthocamptidae

(Claus, 1863)

B4DE7B68-4165-54BB-B1A6-BD3DC95C1D5B

Figs 1 , 2 , 3 , 4 , 5 , 6 , 7 , 8 , 9

B. (B.) minutus vejdovskyiformis Thallwitz, 1916: 238. syn. nov.

##### Material examined.

Estonia • 2 ♀♀ dissected on three slides (BP 546/1-a, BP 546/1-b, BP 546/2); 1 ♂ on one slide (BP 546/3); 9 ♀♀ and 5 ♂♂ undissected preserved in 4% formalin (retained in the collection of the first author); Võrtsjärv Lake; 58.180888°N, 26.089441°E; 25 Sep. 2007; E. Fefilova leg; BP 546.

##### Supplementary description.

**Figure 1.**
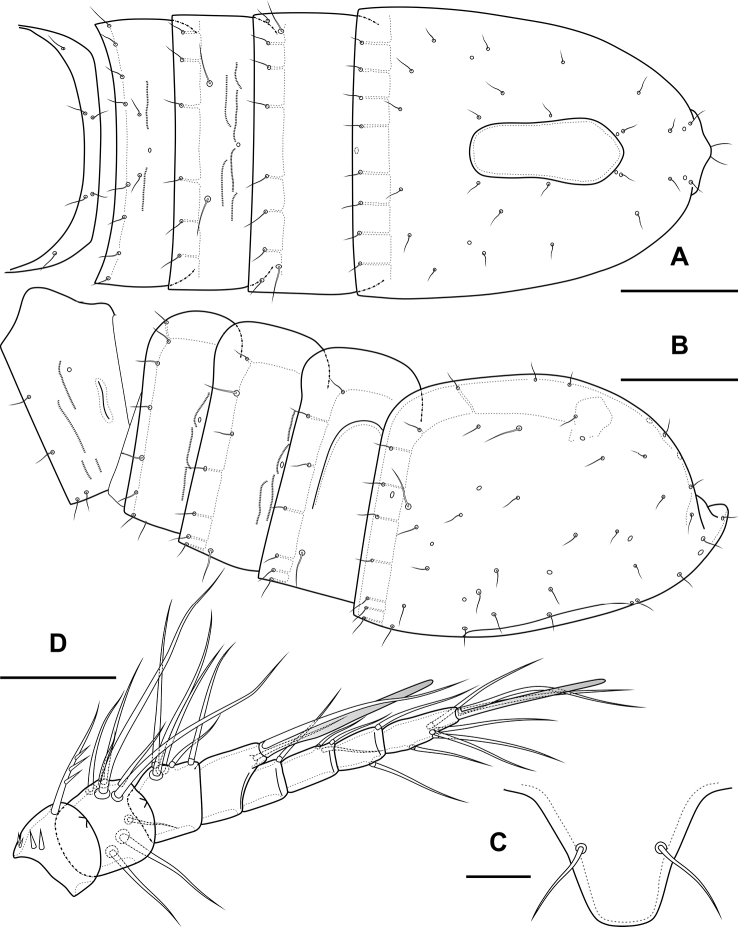
*Bryocamptusminutus*, female **A** cephalothorax and thoracic somites, dorsal **B** cephalothorax and thoracic somites, lateral **C** rostrum **D** antennule. Scale bars: 50 µm (**A, B**); 5 µm (**C**); 25 µm (**D**).

Female. Body subcylindrical. Total body length from anterior margin of rostrum to posterior margin of caudal rami: 484 µm (*n* = 1). Cephalothorax (Fig. 1A, B; Appendix 1) wider than remaining somites, length 151 µm, largest width 124 µm. Naupliar eye not observed. Rostrum (Fig. 1C) small, fused with cephalothorax, with squared end, with one pair of sensillae. Posterior margin of cephalothorax and all pedigerous somites smooth.

Cephalothorax (Fig. 1A, B; Appendix 1) with dumbbell-shaped dorsal window, 10 pairs of pores, seven pairs of sensillae of central group (group C), 13 pairs of sensillae of marginal group (group P) and 20 pairs of ungrouped sensillae (in Table 4 and in Appendix 1 marked as L). Second pedigerous somite with lateral windows, dorsal unpaired pore, lateral pair of pores and eight pairs of sensillae. Third pedigerous somite with dorsal unpaired pore, lateral pair of pores and eight pairs of sensillae. Fourth pedigerous somite with dorsal unpaired pore, lateral pair of pores and eight pairs of sensillae. Fifth pedigerous somite with lateral pair of pores and four pairs of sensillae.

**Figure 2.**
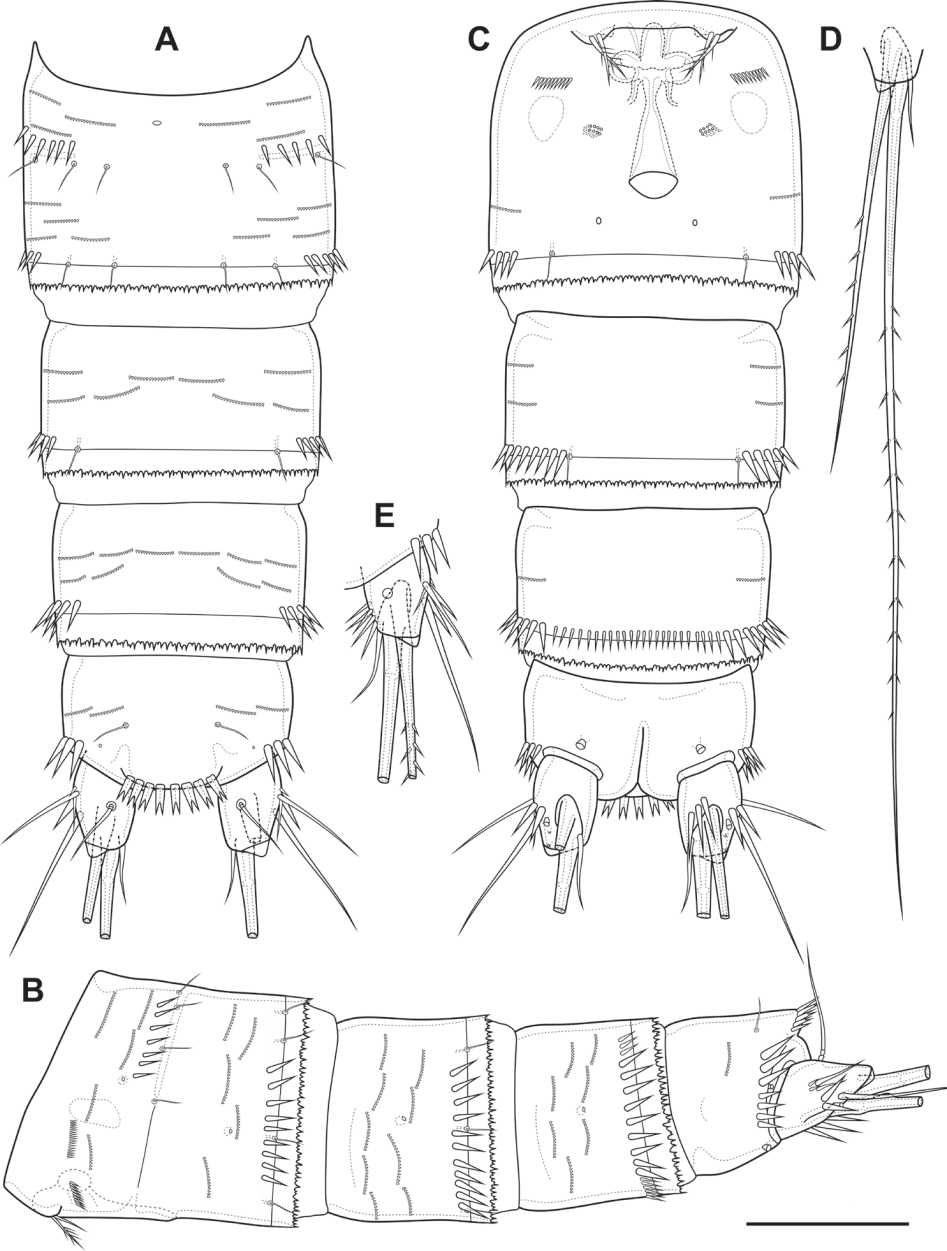
*Bryocamptusminutus*, female **A** abdomen, dorsal **B** abdomen, lateral **C** abdomen, ventral **D** caudal setae, dorsal **E** abnormal caudal ramus, dorsal. Scale bar: 50 µm.

Abdomen (Fig. 2A–C) consisting of genital-double somite, two free abdominal somites and anal somite with caudal rami. All somites except anal somite on posterior margin serrated, on surface with spinular rows. Genital-double somite consists of last thoracic somite and first abdominal somite; longer than wide; anterior part with four pairs of sensillae, dorsal unpaired pore, lateral paired pores, ventro-lateral and lateral rows of spinules; posterior part with four pairs of sensillae, pairs of ventral and lateral pores and lateral rows of spinules.

P6 (Fig. 2C) fused with somite with one pinnate and one naked setae. Genital field (Fig. 2C) long, laterally with eight-pore sieves; copulatory pore displaced to posterior part of somite, copulatory duct chitinised with two additional tubes, extending proximally to pair of labyrinthic rounded ducts and one chitinised unpaired duct.

Second abdominal somite with three pairs of sensillae, pair of lateral pores; on posterior margin with lateral row of large spinules. Third abdominal somite with pair of lateral pores, on posterior margin with lateral row of large spinules and ventral row of small spinules. Anal somite with one pair of sensillae, ventral pair of large pores, lateral pair of pores, dorsal dots near base of caudal rami and lateral spinules. Anal operculum semilunar, with eight long bifid spinules.

Caudal rami (Fig. 2A–E). Length/width ratio 1.6, with three ventral pores; with rows of spinules on ventral side at base of seta IV and rows spinules at base of setae II and III. Seta I small, located near seta II. Setae IV, V and VI displaced to ventral side of caudal ramus. Apical seta IV (Fig. 2D) unipinnate, with “helle Stelle” and massive dorsal bulb located distally “helle Stelle”. Apical seta V long, bipinnate, with “helle Stelle”. Seta VII triarticulated (Fig. 2B).

Antennule (Fig. 1D) 8-segmented. Segment 1 short, with one pinnate seta and two rows of spinules. Other segments with bare setae. Segment 4 with fused basally seta and aesthetasc. Distal segment with acrothek consisting of aesthetasc and two setae fused basally. Armature formula: 1-[1],2-[9],3-[5],4-[1+(1+ae)],5-[1],6-[3],7-[2],8-[5+acr].

**Figure 3.**
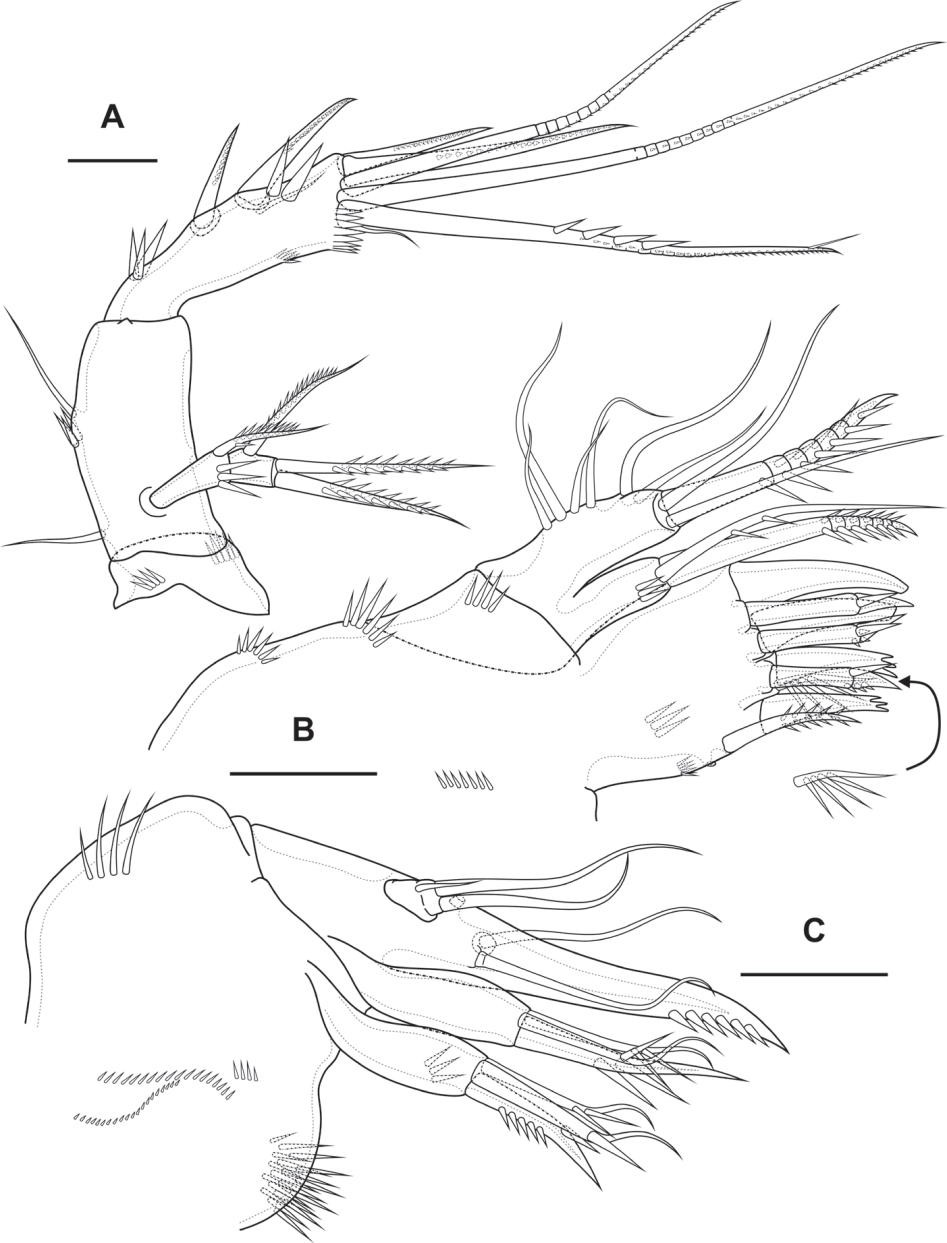
*Bryocamptusminutus*, female **A** antenna **B** maxillule **C** maxilla. Scale bars: 10 µm.

Antenna (Fig. 3A) with allobasis. Coxa with two rows of spinules. Allobasis with two naked setae and one spinular row at base of endopodal seta. Free endopodal segment with two lateral rows of big spinules, with two spinulose spines and slender seta; distally with two rows of spinules; apically with three geniculate setae, two long spines and one small accessory seta; outermost geniculate seta fused basally to small seta. Exopod two-segmented; first segment with one pinnate seta and row of spinules; second segment with three pinnate setae.

**Figure 4.**
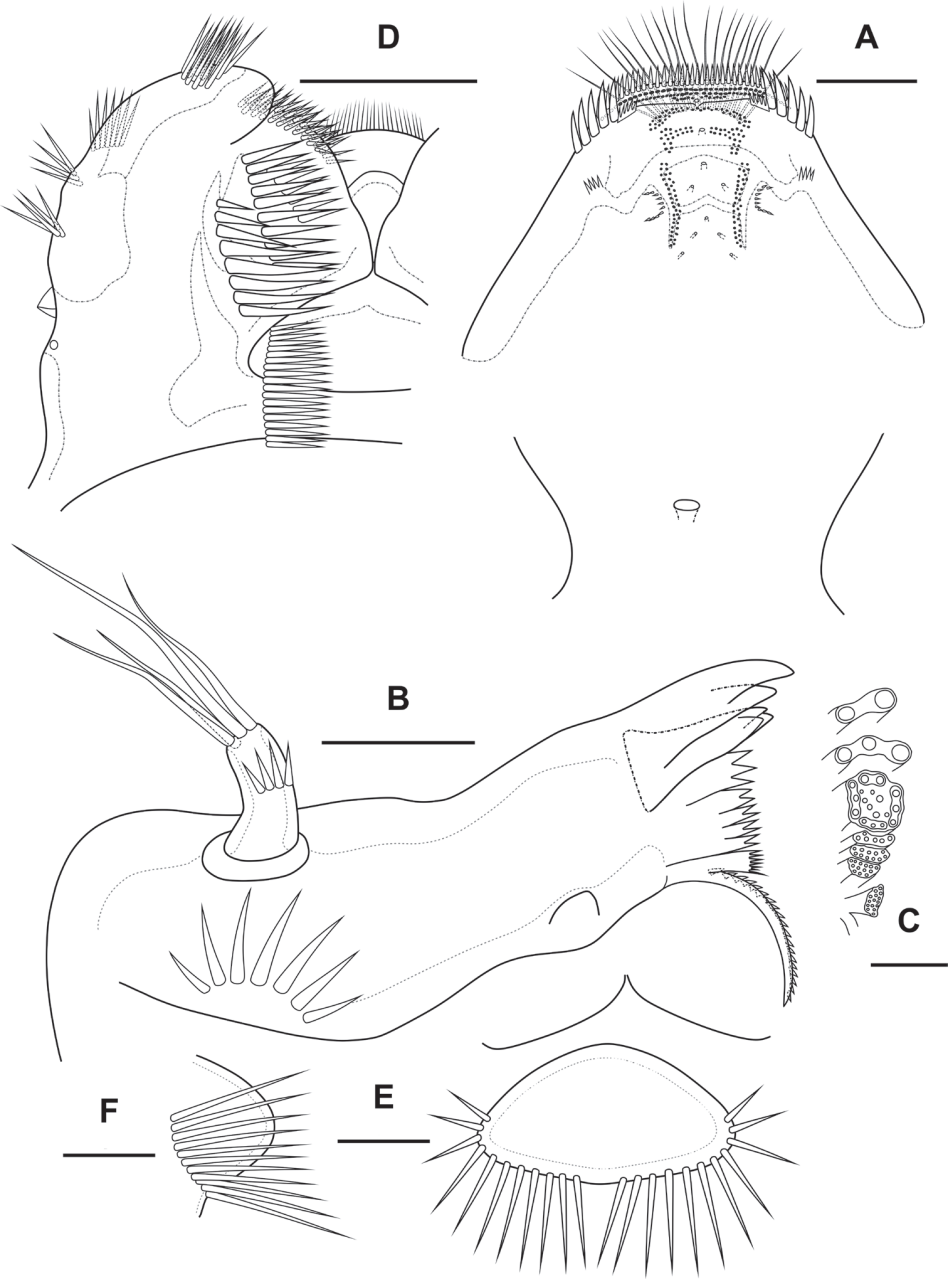
*Bryocamptusminutus*, female **A** labrum, posterior (black dots is bases of spinules) **B** mandible **C** scheme of teeth of mandibular gnathobase **D** paragnaths, anterior **E** cuticular process between maxillipeds and P1, ventral **F** cuticular process between maxillipeds and P1, lateral. Scale bars: 10 µm (**A, B, D**); 5 µm (**C, E, F**).

Labrum (Fig. 4A). On outer side with row of thin setules and large proximal pore. Distal margin with lateral rows of robust spinules, rows of fused spinules into comb and three rows of small spinules. On inner side medially with four unpaired pores, three pared pores, with lateral spinular row, semicircular spinular row and groups of thin setules.

Mandible (Fig. 4B, C). Coxa with spinules proximally. Gnathobase with pars incisiva, lacinia mobilis, complex dental battery and spinulose seta; pars incisiva two-pointed; lacinia mobilis three-pointed. Dental battery (Fig. 4C) consisting of five fused blocks of small short teeth, inner of which fused at base with seta. Pars molaris sharply edged. Palp one-segmented, with medial spinular row and four apical setae.

Paragnaths (Fig. 4D) with paired lateral lobes and unpaired posterior rounded lobe. Lateral lobes wrapped in distal part forming “pocket”; proximally with lateral pore (probably); on outer side with four groups of long spinules; on inner side with three-four rows of spinules; on anterior side with three medial rows of strong spinules and proximal row of spinules.

Maxillule (Fig. 3B). Praecoxa with two rows of slender spinules on outer edge and one row of spinules on posterior side. Praecoxal arthrite medially with two rows of spinules and one proximal pore; distally with one simple strong spine, three strong spines with pectinate end, three biarticulate spines, one proximal bipinnate seta and one thin seta with long spinules. Coxa with row of spinules, coxal endite with one weakly pinnate and one spinulose geniculate setae. Basis with two subdistal setae and three distal setae, one of which spinulose and geniculate. Endopod and exopod incorporated into basis, each represented by two naked setae.

Maxilla (Fig. 3C). Basis with several rows of spinules on outer and inner edge as figured, with two endites. Proximal endite with spinular row, one spinulose spine and two pinnate setae, distal endite with one strong pinnate seta and two thin pinnate setae. Proximal endopodal segment with two setae, outer tube pore and massive distal claw. Distal endopodal segment with three naked setae, one of which proximal and small.

**Figure 5.**
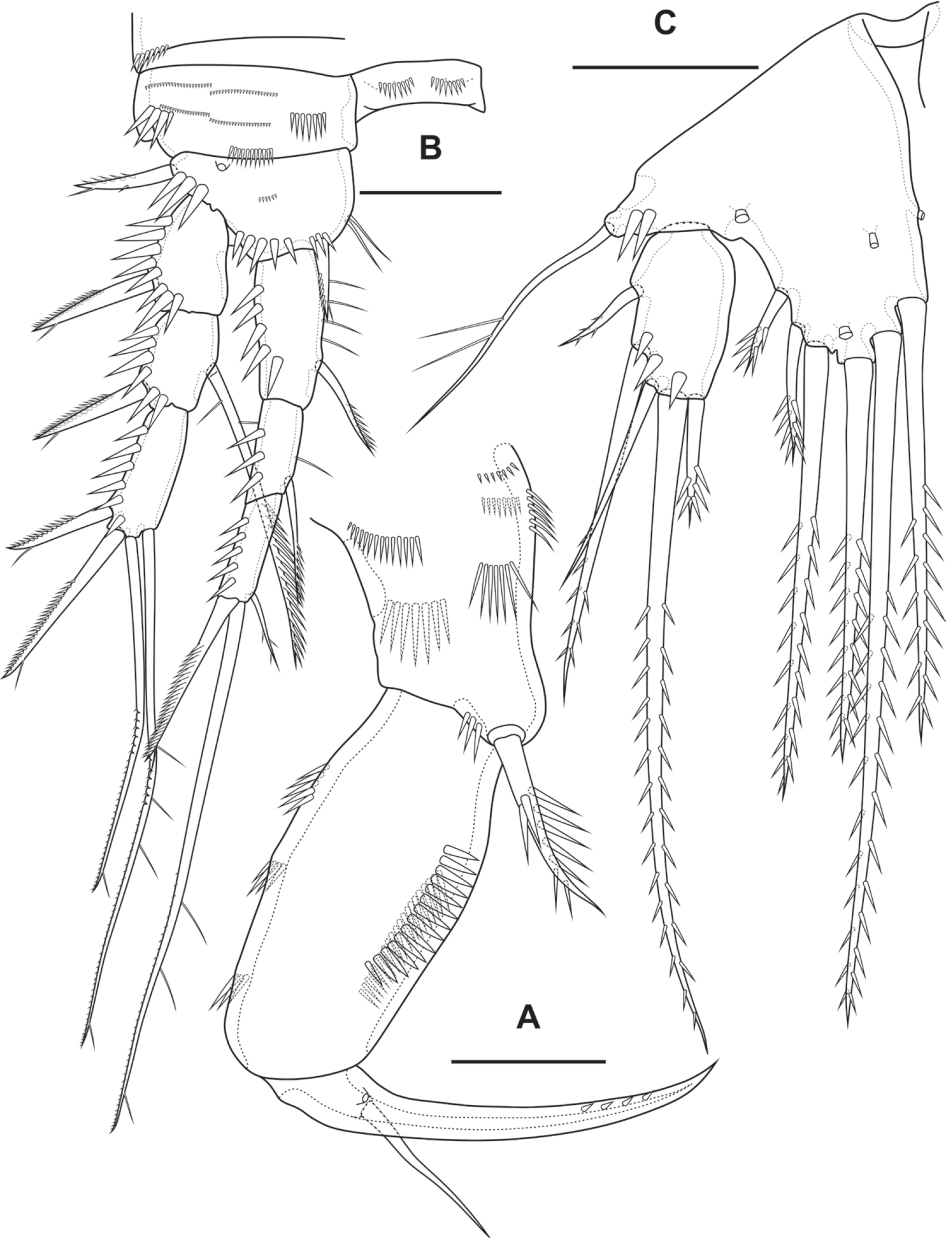
*Bryocamptusminutus*, female **A** maxilliped **B**P1, anterior **C**P5, anterior. Scale bars: 10 µm (**A**); 25 µm (**B, C**).

Maxilliped (Fig. 5A) subchelate. Syncoxa elongated with several rows of spinules as figured, distally with one pinnate seta. Basis with two rows of large spinules on anterior and posterior sides and three outer rows of small spinules. Endopod on posterior side with one seta, on anterior side with small protuberance, probably tube pore. Endopodal claw elongated, with row of small spinules.

Cuticular process between maxillipeds and P1 (Fig. 4E, F) in height approximately same as in length, with long spinules, ten spinules on each side. Spinules encircle from anterior-lateral margin to posterior margin.

P1 (Fig. 5B; Table 1) with three-segmented rami. Praecoxa with outer spinular row. Coxa rectangular, with seven spinular rows, four of which consisting of little spinules. Intercoxal sclerite wide, with one paired spinular rows. Basis with proximal pore, medial row of small spinules, rows of spinules at base of endopod and exopod, row of spinules at base of inner seta, inner row of spinules; with inner and outer strong spines. All endopodal and exopodal segments with outer spinules. First exopodal segment with one outer spinulose spine; second segment with inner pectinate seta and outer spinulose spine; third exopodal segment with two outer spinulose spines and two apical slender geniculate setae. Endopod longer than exopod. First endopodal segment reaching middle of second exopodal segment, with inner pectinate seta and inner spinular row; second endopodal segments with one inner pectinate seta, third segment with outer spinulose spine, apical long geniculate seta and inner small seta.

**Table 1.** P1–P4 armature of examined specimens of *Bryocamptusminutusminutus*.

	Female endopod	Male endopod	Exopod
P1	1; 1; 1,1,1	1; 1; 1,1,1	0; 1; 0,2,2
P2	1; 1; 1,2,1	1; 2,2,0	0; 1; 1,2,3
P3	1; 1; 2,2,1	1; 1+ ap; 2,2,0	0; 1; 2,2,3
P4	1; 2,2,1	0; 1,2,1	0; 1; 2,2,2

**Figure 6.**
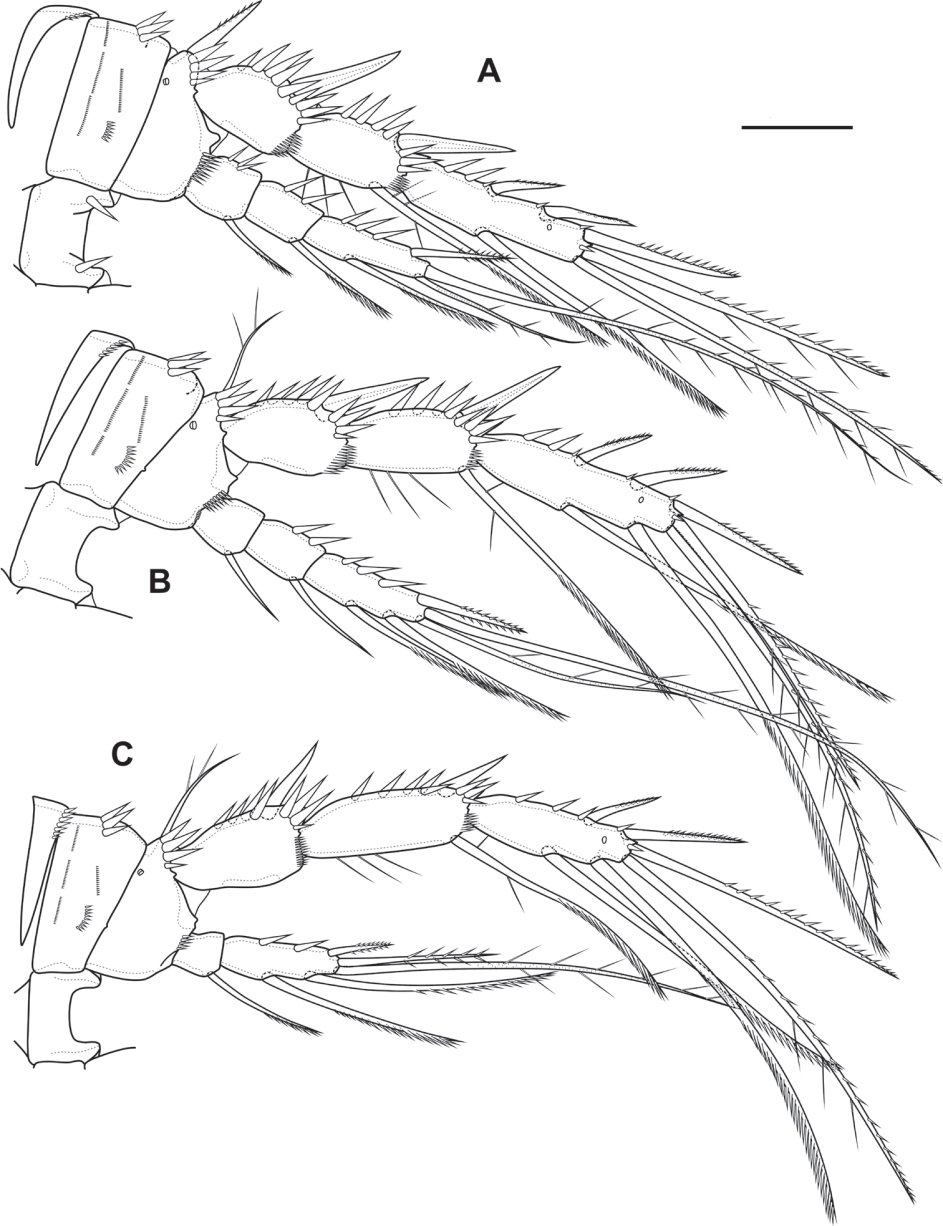
*Bryocamptusminutus*, female **A**P2, anterior **B**P3, anterior **C**P4, anterior. Scale bar: 25 µm.

P2 (Fig. 6A; Table 1). Praecoxa with row of spinules. Coxa with one lateral row of large spinules and five rows of spinules on anterior side. Intercoxal sclerite with two large spinules. Basis with proximal pore, rows of spinules at base of endopod and exopod; with outer spine. All endopodal and exopodal segments with outer spinules. Exopod three-segmented; first exopodal segment with outer naked spine, apically with frill; second segment with outer naked spine, inner pectinate seta, inner slender spinules and apical frill; third segment with pore, three outer spinulose spines, two apical setae and one inner pectinate seta. Endopod three-segmented; first and second segments with inner seta; third segment with outer spinulose spine, two apical pinnate setae and one inner pectinate seta.

P3 (Fig. 6B; Table 1). Praecoxa with spinular row. Coxa with one lateral row of large spinules and five rows of spinules on anterior side. Intercoxal sclerite without spinules. Basis with outer seta, proximal pore, rows of spinules at base of endopod and exopod. Exopod three-segmented; first exopodal segment with outer naked spine, outer spinules, apically with frill; second segment with outer naked spine, outer spinules, inner pectinate seta, inner slender spinules and apical frill; third segment with pore, three outer spinulose spines, two apical setae and two inner pectinate setae. Endopod three-segmented; first and second segments with inner seta, second segment with outer spinules; third segment with outer spinules, outer spinulose spine, two apical pinnate setae and two inner pectinate setae.

P4 (Fig. 6C; Table 1). Praecoxa with spinular row. Coxa with one lateral row of large spinules and five rows of spinules on anterior side. Intercoxal sclerite without spinules. Basis with outer seta, proximal pore, rows of spinules at base of endopod and exopod. Exopod three-segmented; first exopodal segment with outer naked spine, outer spinules, apically with frill; second segment with outer naked spine, outer spinules, inner pectinate seta, inner slender spinules and apical frill; third segment with pore, two outer spinulose spines, two apical setae and two inner pectinate setae. Endopod two-segmented; first segment with inner seta, second segment with outer spinules, outer spinulose spine, two apical pinnate setae and two inner pectinate setae.

P5 (Fig. 5C) with separate right and left baseoendopods. Baseoendopod reaching ~ 1/2 of exopodal segment; with four pores, spinular row at base of outer seta; outer seta of basis pinnate, long. Endopodal lobe with four long bipinnate setae and two short bipinnate setae V and VI; with small process that may be pore between setae III and IV. Exopod with inner short pinnate seta, long apical pinnate seta, naked subapical seta and two pinnate outer setae.

**Figure 7.**
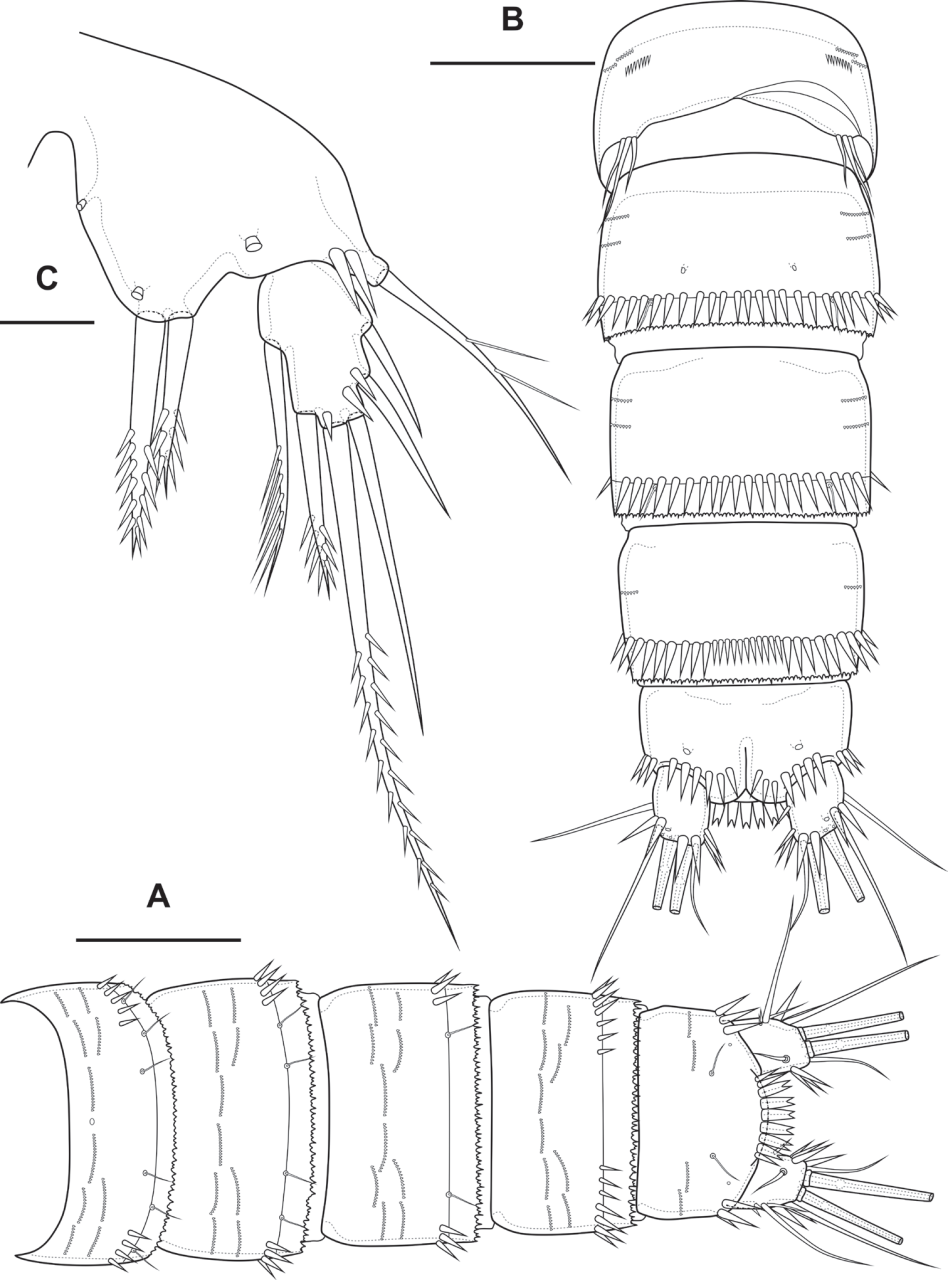
*Bryocamptusminutus*, male **A** abdomen, dorsal **B** abdomen, ventral **C**P5, anterior. Scale bars: 50 µm (**A, B**); 10 µm (**C**).

Male. Sexual dimorphism expressed in the antennule, P2–P6, genital segmentation and ornamentation, shape of caudal rami. Cephalothorax and thoracic somites as in female. P6 (Fig. 7B) two asymmetric flaps fused to the somite, with three naked setae. Differences from female in abdomen structure as follows (Fig. 7A, B): first abdominal somite free; first to third abdominal somites with spinular row encircling somite ventrally and laterally; anal somite with ventral spinules; caudal rami with normal setae IV and V; anal operculum with nine bifid and simple spinules.

**Figure 8.**
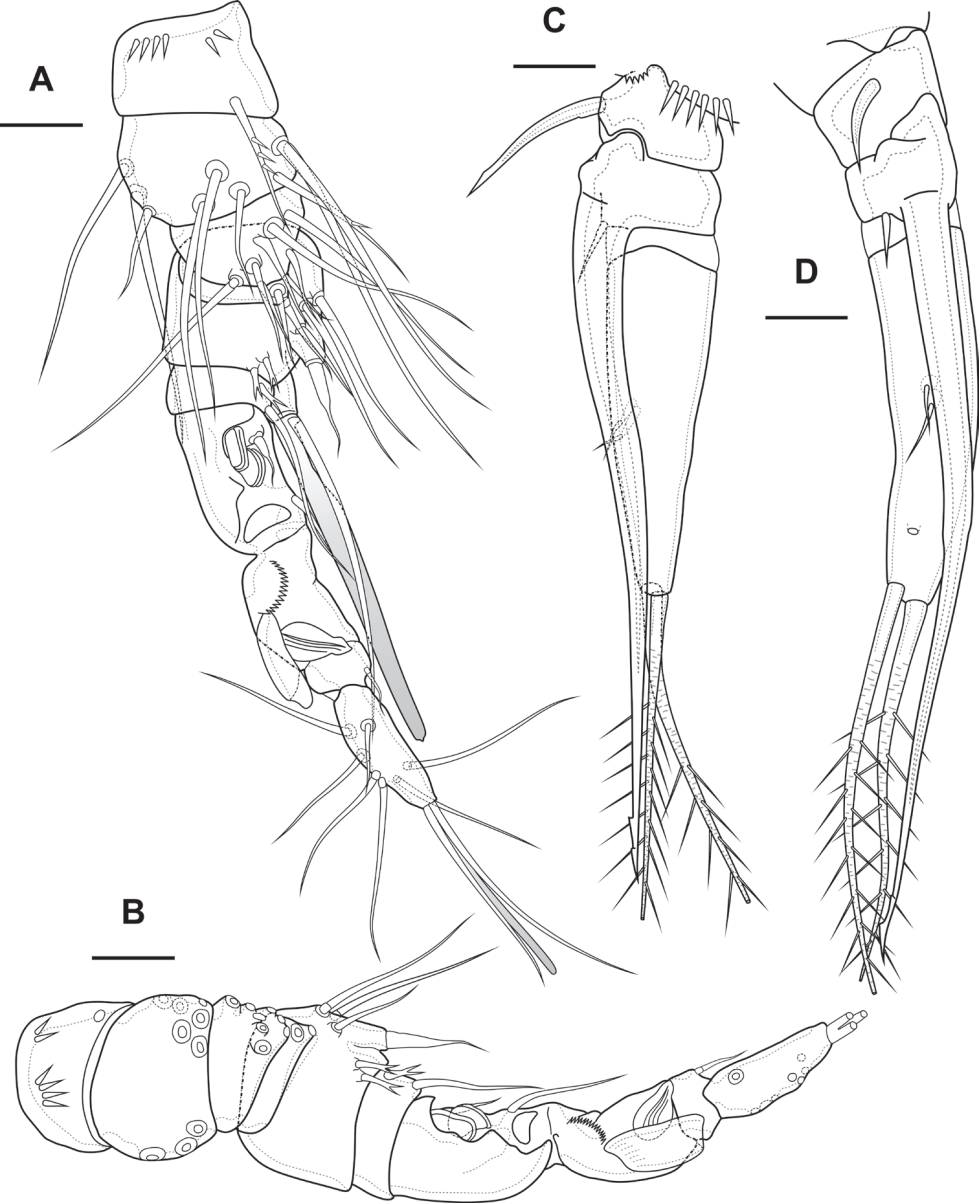
*Bryocamptusminutus*, male **A** antennule, anterior **B** antennule, dorsal **C**P3 endopod, anterior **D**P3 endopod, inner view. Scale bars: 10 µm.

Antennule (Fig. 8A, B) 10-segmented, haplocer with geniculation between segments 7 and 8. Segment 5 with large aestetasc fused at base with long seta, with one strong caudate seta. Segment 7 with articular plate, with one filiform seta, one small caudate seta and with two modified laminar setae. Segment 8 with proximal dentate plate and two strong modified laminar setae. Segment 10 with acrothek consisting of slender aestetasc and two setae. Armature formula: 1-[1],2-[9],3-[8],4-[2],5-[6+(1+ae)],6-[2],7-[2+2 modified],8-[2 modified],9-[1],10-[7+acr].

**Figure 9.**
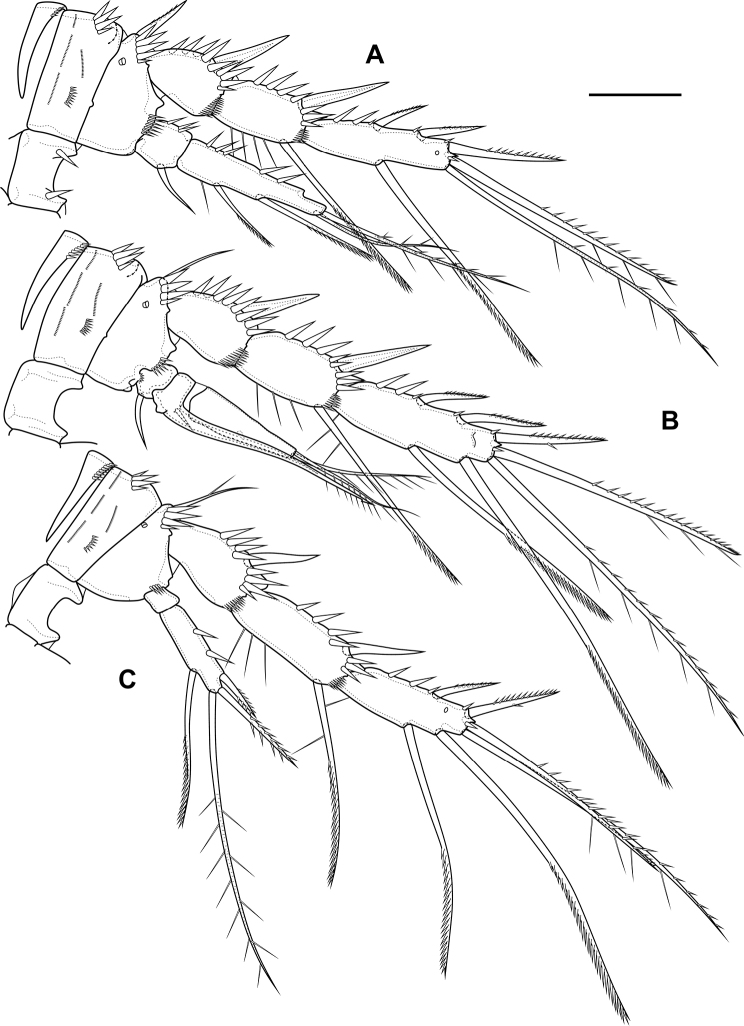
*Bryocamptusminutus*, male **A**P2, anterior **B**P3, anterior **C**P4, anterior. Scale bar: 25 µm.

P2 (Fig. 9A) as in female, except endopod. Endopod two-segmented. First segment with outer spinules and inner seta. Second segment with notch on distal outer margin, outer spinules, two apical pinnate slender setae and two inner pectinate setae.

P3 (Figs 8C, D, 9B): praecoxa, coxa, intercoxal sclerite as in female. Basis as in female, but with inner process. Exopod as in female, but third segment with broad slit-like pore. Endopod three-segmented. First endopodal segment with strong seta. Second endopodal segment with posterior seta and long apophysis with double tip. Third segment with two small inner setae, inner pore and two apical pinnate setae.

P4 (Fig. 9C): praecoxa, coxa, intercoxal sclerite, basis, exopod as in female. Endopod two-segmented; first segment short unarmed; second segment with outer spinules, spinulose spine, outer apical spiniform spinulose seta, inner apical bipinnate seta and inner pectinate seta.

P5 (Fig. 7C) right and left fused medially. Baseoendopod with three pairs of pores, outer spinular row and outer long pinnate seta; endopodal lobe with two strong spinulose apical spines. Exopod with spinules on anterior surface, three naked outer setae, long apical spinulose seta, one inner spinulose seta and one long inner pectinate seta with long setules.

##### Variability.

We found variability in the structure of the caudal rami. Some females have an inner group of long spinules (Fig. 2E).

#### ﻿ Bryocamptus (Bryocamptus) abramovae sp. nov.

Taxon classificationAnimaliaHarpacticoidaCanthocamptidae

68B743E1-F92A-527E-99AB-9271A458A8EE

https://zoobank.org/D2258B3F-4D75-4D53-B4CA-259A0D2F20F0

Figs 10 , 11 , 12 , 13 , 14 , 15 , 16 , 17 , 18

Bryocamptus sp. 2 – Novikov et al. 2021: 271.
Bryocamptus sp. 1 – Novikov and Sharafutdinova 2022: 34.

##### Material.

***Holotype***: Russia • ♀ dissected on two slides; Lena River Delta, Samoylov Island, Ruiba Lake; 72.373003°N, 126.489429°E; depth 1–1.5 m; 23 Aug. 2019; A. Novikov leg; BP 547/1-a, BP 547/1-b. ***Allotype***: Russia •♂ dissected on one slide; collection data as for holotype; BP 547/2. ***Paratypes***: 5 ♀ and 3 ♂ undissected, preserved in 4% formalin; collection data as for holotype; BP 547/4.

##### Additional material.

Russia • 9 ♀♀ and 6 ♂♂ undissected; Lena River Delta, Jangylakh Sise Island, large nameless lake; 72.517921°N, 125.281147°E; 7 Aug. 2019; A. Novikov leg; retained in the collection of the first author.

Russia • 2 ♀♀ undissected; Lena River Delta, Baron Island, small thermokarst lake; 72.550939°N, 126.93597°E; 8 Aug. 2019; A. Novikov leg; retained in the collection of the first author.

Russia • 3 ♀♀ and 1 ♂ undissected; Lena River Delta, Kurungnah Sise Island, Krugloe Lake; 72.468859°N, 126.265658°E; 21 Aug. 2019; A. Novikov leg; retained in the collection of the first author

Russia • 4 ♀♀ and 2 ♂♂ undissected; Vrangel Island, large nameless lake; 70.954443°N, 179.567387°E; 26 Aug. 2021; A. Novichkova leg: retained in the collection of the first author.

##### Description.

**Figure 10.**
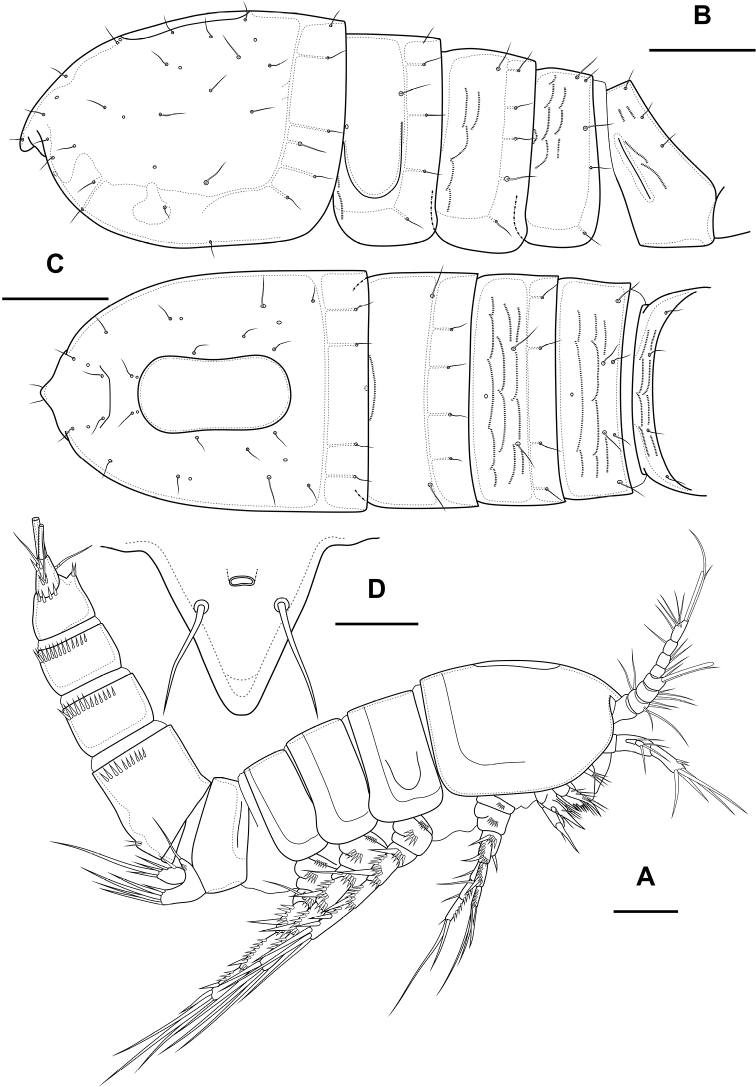
*Bryocamptusabramovae* sp. nov., female **A** habitus, lateral **B** cephalothorax and thoracic somites, dorsal **C** cephalothorax and thoracic somites, lateral **D** rostrum. Scale bars: 50 µm (**A–C**); 5 µm (**D**).

Female (based on holotype and paratypes). Body subcylindrical (Fig. 10A). Total body length from anterior margin of rostrum to posterior margin of caudal rami: 586 µm (*n* = 1). Cephalothorax (Fig. 10B, C; Appendix 1), wider as remaining somites, length 152 µm, largest width 113 µm. Naupliar eye red. Rostrum (Fig. 10D) small, fused with cephalothorax, with rounded end, with one pair of sensillae and pore located proximal to sensillae. Posterior margin of cephalothorax and all pedigerous somites smooth.

Cephalothorax (Fig. 10B, C; Appendix 1) with dumbbell-shaped dorsal window, seven pairs of pores, seven pairs of sensillae of central group (group C), eight pairs of sensillae of marginal group (group P) and 13 pairs of ungrouped sensillae (in Table 4 and in Appendix 1 marked as L). Second pedigerous somite with lateral windows, dorsal unpaired pore, lateral pair of pores and six pairs of sensillae. Third pedigerous somite with dorsal unpaired pore and six pairs of sensillae. Fourth pedigerous somite with dorsal unpaired pore and five pairs of sensillae. Fifth pedigerous somite with three pairs of sensillae.

**Figure 11.**
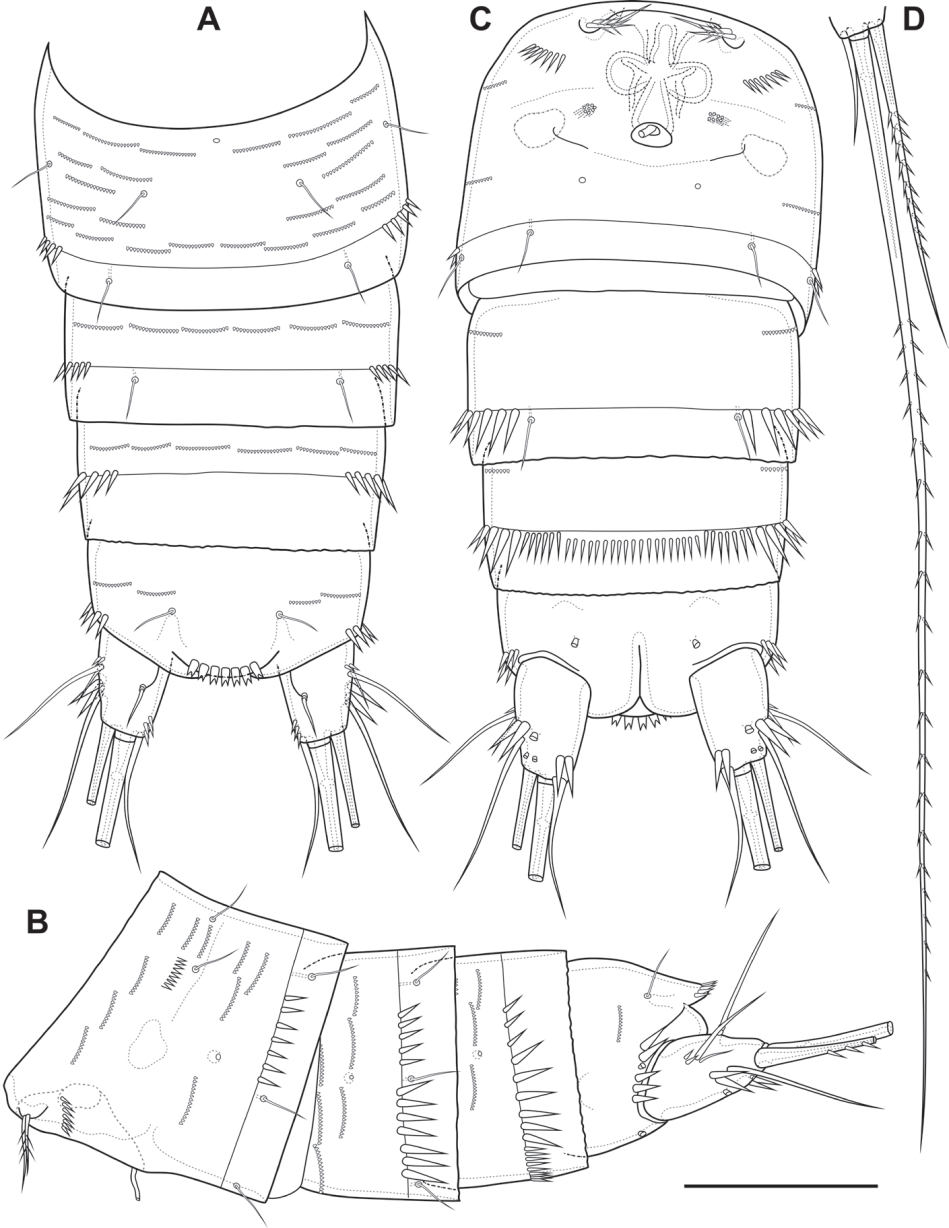
*Bryocamptusabramovae* sp. nov., female **A** abdomen, dorsal **B** abdomen, lateral **C** abdomen, ventral **D** caudal setae, dorsal. Scale bar: 50 µm.

Abdomen (Fig. 11A–C) consisting of genital-double somite, two free abdominal somites and anal somite with caudal rami. All somites except anal somite slightly wavy posterior margin, on surface with spinular rows. Genital-double somite consists of last thoracic somite and first abdominal somite; wider than long; anterior part with two pairs of sensillae, dorsal unpaired pore, ventro-lateral row of spinules; posterior part with three pairs of sensillae, pairs of ventral and lateral pores and lateral rows of spinules.

P6 (Fig. 11C) fused with somite with one pinnate and one naked setae. Genital field (Fig. 11C) short, laterally with eight-pore sieves; copulatory pore located medially, copulatory duct chitinised with two additional tubes, extending proximally to pair of labyrinthic rounded ducts and one chitinised unpaired duct.

Second and third abdominal somites as in *B.minutus*. Anal somite with one pair of sensillae, ventral pair of large pores, lateral pair of pores and lateral spinules. Anal operculum semilunar, with seven short bifid spinules.

Caudal rami (Fig. 11A–D). Length/width ratio 1.6, with three ventral pores; with rows of spinules on ventral and dorsal side at base of seta VI and rows spinules at base of setae II and III. Seta I small, located near seta II. Apical seta IV (Fig. 11D) bipinnate, without “helle Stelle”. Apical seta V long, bipinnate, with “helle Stelle”. Seta VI with wide base (Fig. 11C). Seta VII triarticulated (Fig. 11B).

**Figure 12.**
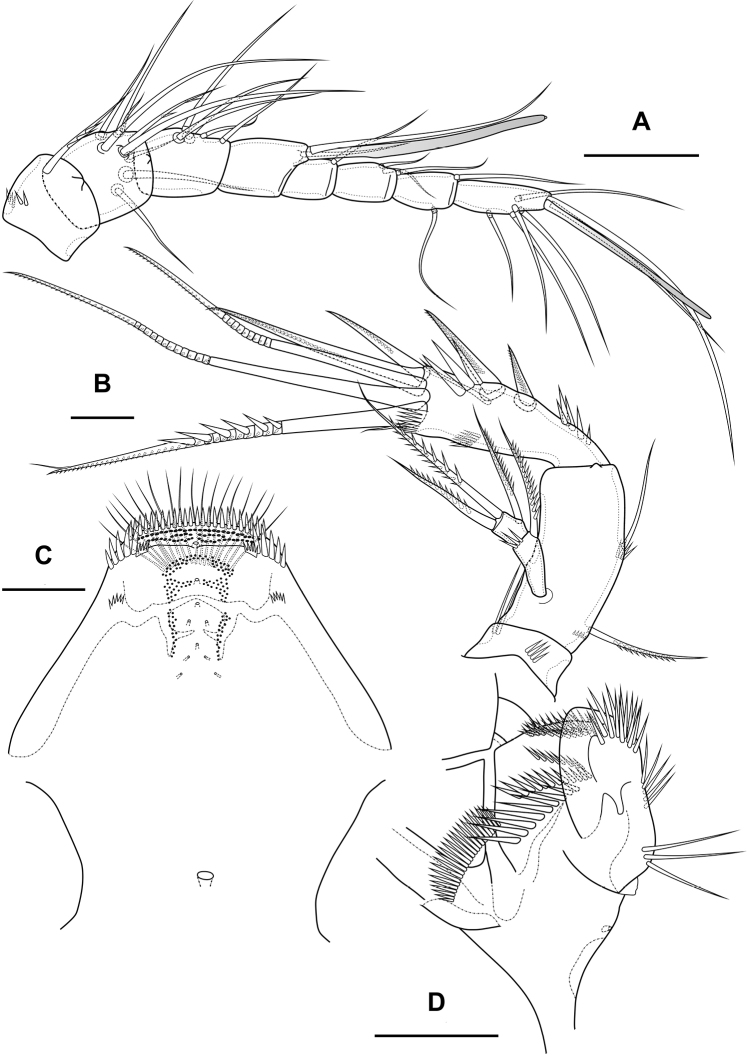
*Bryocamptusabramovae* sp. nov., female **A** antennule **B** antenna **C** labrum, posterior (black dots is bases of spinules) **D** paragnaths, anterior. Scale bars: 25 µm (**A**); 10 µm (**B–D**).

Antennule (Fig. 12A) similar to that of *Bryocamptusminutus*. Differences expressed in more elongated segments, especially 3^th^ and 4^th^ segments; one of setae on segment 2 pinnate. Armature formula: 1-[1],2-[9],3-[5],4-[1+(1+ae)],5-[1],6-[3],7-[2],8-[5+acr].

Antenna (Fig. 12B) similar to that of *Bryocamptusminutus*. Allobasis and free endopodal segment slightly more elongated. Inner spinular row on coxa with extremely long spinules. Allobasis with proximal outer spinular row, basal seta pinnate.

Labrum (Fig. 12C) similar to that of *Bryocamptusminutus*, but without semicircular spinular row on inner side.

**Figure 13.**
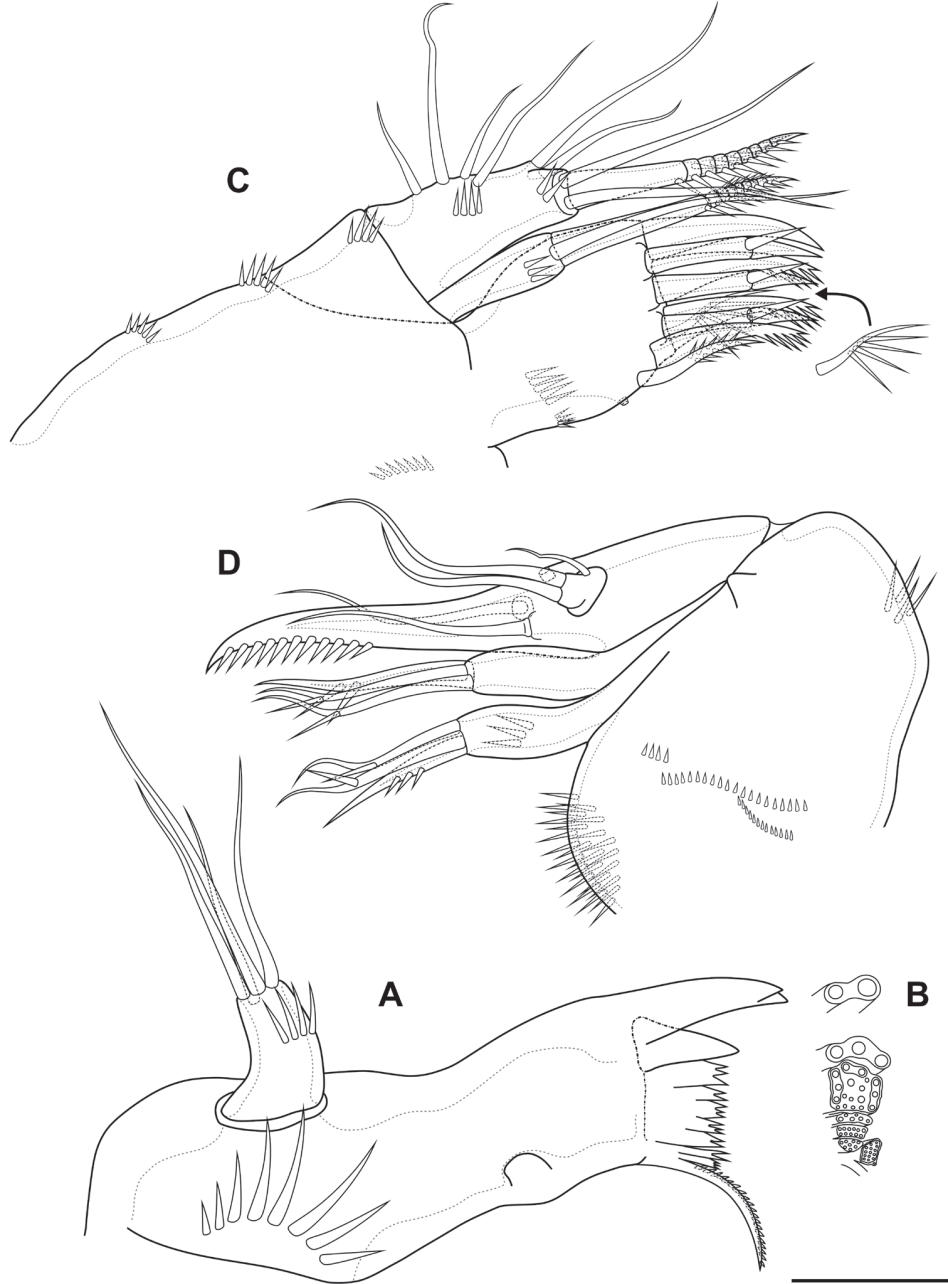
*Bryocamptusabramovae* sp. nov., female **A** mandible **B** scheme of teeth of mandibular gnathobase **C** maxillule **D** maxilla. Scale bar: 10 µm.

Mandible (Fig. 13A, B) similar to that of *Bryocamptusminutus*. The palp is shortened.

Paragnaths (Fig. 12D) similar to that of *Bryocamptusminutus*, with only three lateral groups of spinules and with a more well-defined pocket.

Maxillule (Fig. 13C) similar to that of *Bryocamptusminutus*. Basis with two groups of spinules.

Maxilla (Fig. 13D) as in *Bryocamptusminutus*, only with slight differences in length and armature of setae.

**Figure 14.**
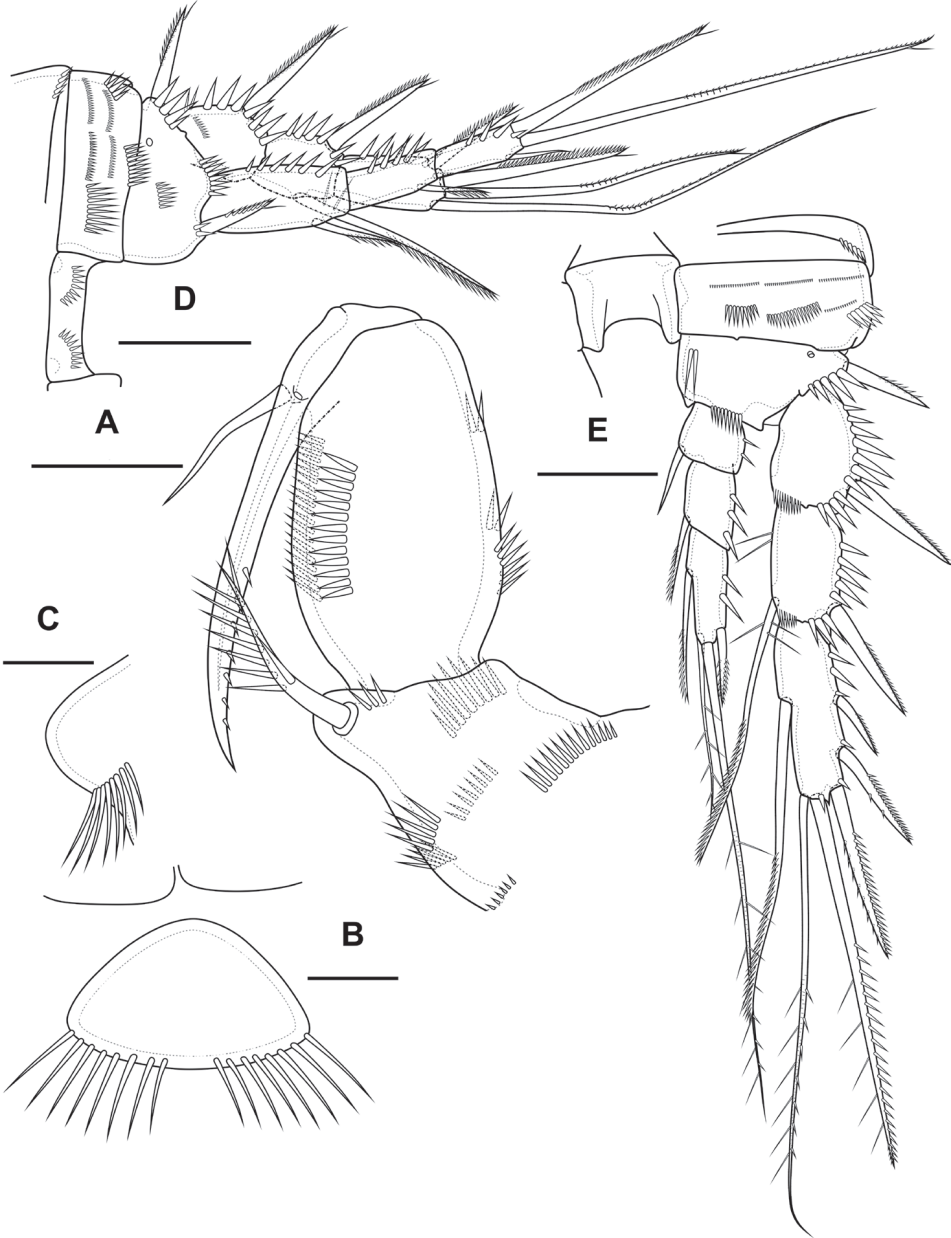
*Bryocamptusabramovae* sp. nov., female **A** maxilliped **B** cuticular process between maxillipeds and P1, ventral **C** cuticular process between maxillipeds and P1, lateral **D**P1, anterior **E**P2, anterior. Scale bars: 10 µm (**A**); 5 µm (**B, C**); 25 µm (**D, E**).

Maxilliped (Fig. 14A) similar to that of *Bryocamptusminutus*. Differences are only in shorter syncoxa and basis.

Cuticular process between maxillipeds and P1 (Fig. 14B, C) in height approximately same as in length, with long spinules, seven spinules on each side. Spinules on posterior margin.

P1 (Fig. 14D; Table 2) similar to that of *Bryocamptusminutus*. Basis without inner spinules. First exopodal segment with row of small spinules on anterior side. First endopodal segment reaching end of second exopodal segment. First and second endopodal segments with smooth inner side. Differences also noticeable in shorter exopodal and endopodal segments and larger spinules on coxa and basis.

**Table 2.** P1 – P4 armature of *Bryocamptusabramovae* sp. nov.

	Female endopod	Male endopod	Exopod
P1	1; 1; 1,1,1	1; 1; 1,1,1	0; 1; 0,2,2
P2	1; 1; 1,2,1	1; 2,2,0	0; 1; 1,2,2-3
P3	1; 1; 2,2,1	1; 1+ ap; 2,2,0	0; 1; 2,2,2-3
P4	1; 2,2,1	0; 0,2,1	0; 1; 2,2,2-3

P2 (Fig. 14E; Table 2). Praecoxa with row of spinules. Coxa with one lateral row of large spinules, two anterior rows of large spinules and four anterior rows of small spinules. Intercoxal sclerite naked. Basis with proximal pore, inner group of long spinules, rows of spinules at base of endopod and exopod; with outer spine. All endopodal and exopodal segments with outer spinules. Exopod three-segmented; first exopodal segment with outer spinulose spine, apically with frill; second segment with outer spinulose spine, inner pectinate seta, inner slender spinules and apical frill; third segment with three outer spinulose spines, two apical setae and one inner pectinate seta. Endopod three-segmented; first and second segments with inner seta; third segment with outer spinulose spine, two apical pinnate setae and one inner pectinate seta.

**Figure 15.**
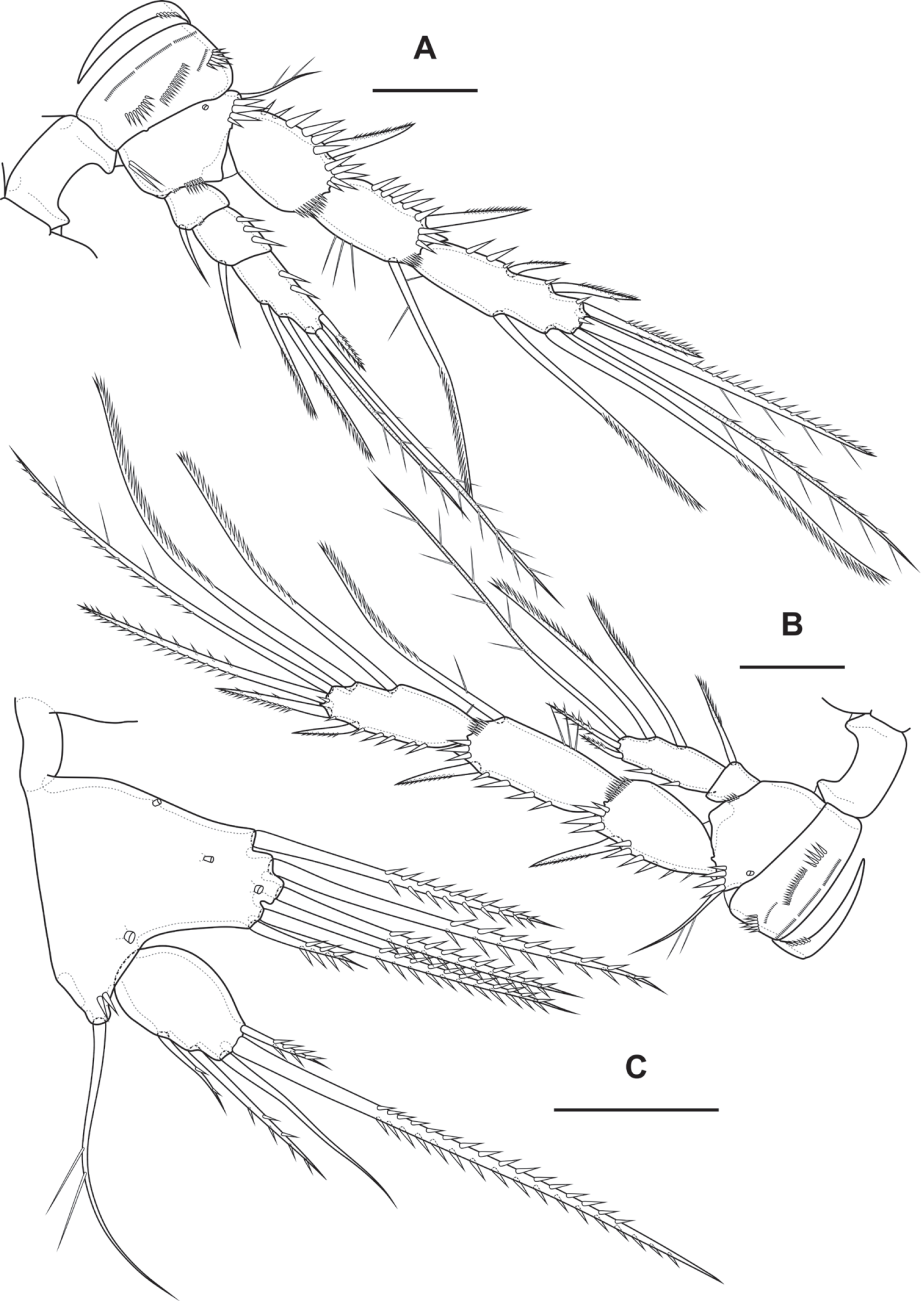
*Bryocamptusabramovae* sp. nov., female **A**P3, anterior **B**P4, anterior **C**P5, anterior. Scale bars: 25 µm.

P3 (Fig. 15A; Table 2). Praecoxa with spinular row. Coxa with one lateral row of large spinules, two anterior rows of large spinules and four anterior rows of small spinules. Intercoxal sclerite without spinules. Basis with outer seta, proximal pore, inner group of long spinules and rows of spinules at base of endopod and exopod. Exopod three-segmented; first exopodal segment with outer spinulose spine, outer spinules, apically with frill; second segment with outer spinulose spine, outer spinules, inner pectinate seta, inner slender spinules and apical frill; third segment with three outer spinulose spines, two apical setae and two inner pectinate setae. Endopod three-segmented; first and second segments with inner seta, second segment with outer spinules; third segment with outer spinules, outer spinulose spine, two apical pinnate setae and two inner pectinate setae.

P4 (Fig. 15B; Table 2). Praecoxa with spinular row. Coxa with one lateral row of large spinules, two anterior rows of large spinules and four anterior rows of small spinules. Basis with outer seta, proximal pore, rows of spinules at base of endopod and exopod. Exopod three-segmented; first exopodal segment with outer spinulose spine, outer spinules, apically with frill; second segment with outer spinulose spine, outer spinules, inner pectinate seta, inner slender spinules and apical frill; third segment with two outer spinulose spines, two apical setae and two inner pectinate setae. Endopod two-segmented; first segment with inner pectinate seta, second segment with outer spinules, outer spinulose spine, apical spiniform spinulose seta, apical pinnate seta and two inner pectinate setae.

P5 (Fig. 15C) with separate right and left baseoendopods. Baseoendopod reaching ~ 2/3 of exopodal segment; with four pores, spinular row at base of outer seta; outer seta of basis pinnate, long. Endopodal lobe with four long bipinnate setae and one short bipinnate seta V; with small process that may be pore between setae III and IV. Exopod inner thin pinnate seta, long apical pinnate seta, naked subapical seta and two pinnate outer setae.

**Figure 16.**
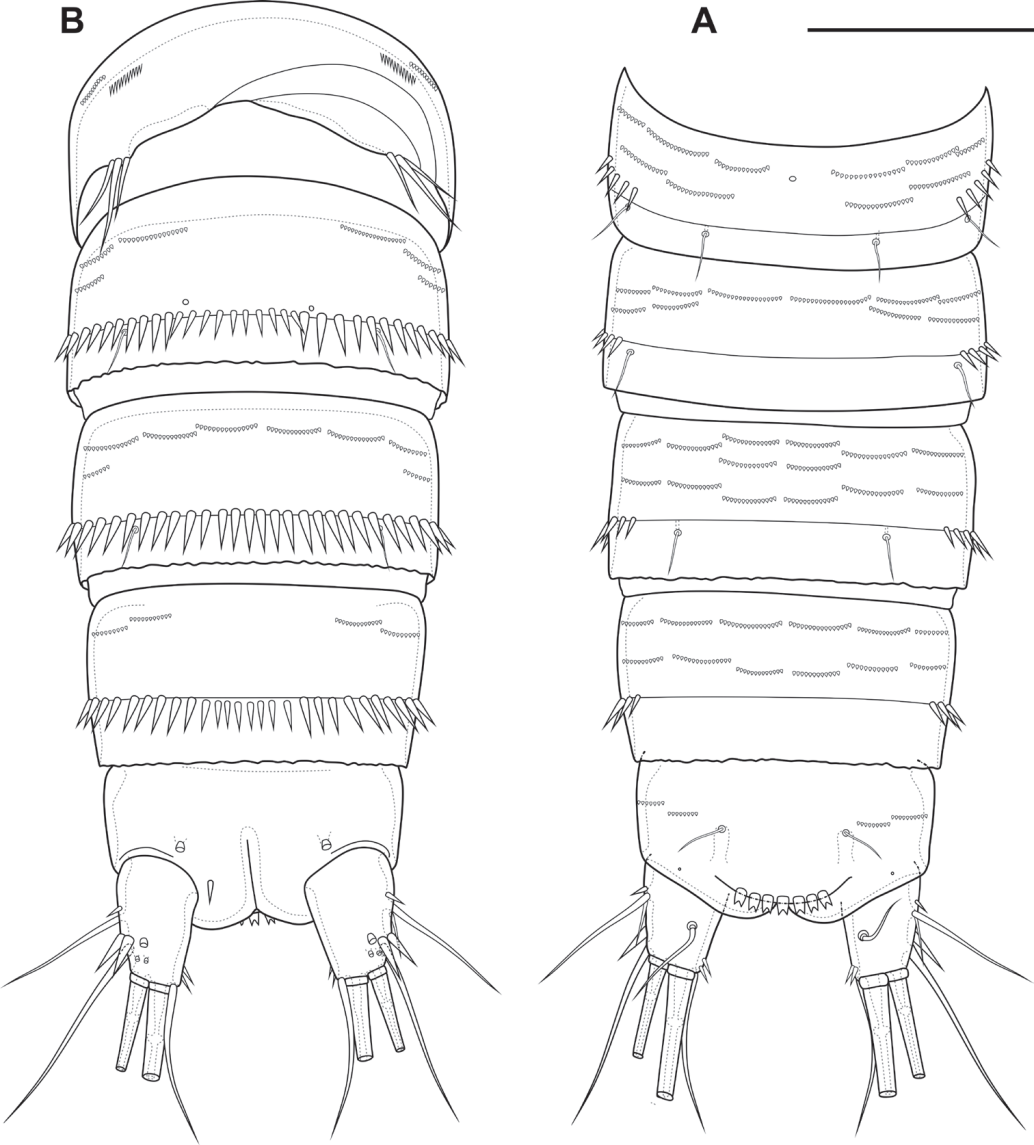
*Bryocamptusabramovae* sp. nov., male **A** abdomen, dorsal **B** abdomen, ventral. Scale bar: 50 µm.

Male. Sexual dimorphism expressed in the antennule, P2–P6, genital segmentation and ornamentation, shape of caudal rami. Cephalothorax and thoracic somites as in female. P6 (Fig. 16B) two asymmetric flaps fused to the somite, with three naked setae. Differences from female in abdomen structure as follows (Fig. 16A, B): first abdominal somite free; first to third abdominal somites with spinular row encircling somite ventrally and laterally; anal somite with ventral spinule and without lateral spinules; caudal rami without ventral spinules; seta IV with “helle Stelle”.

**Figure 17.**
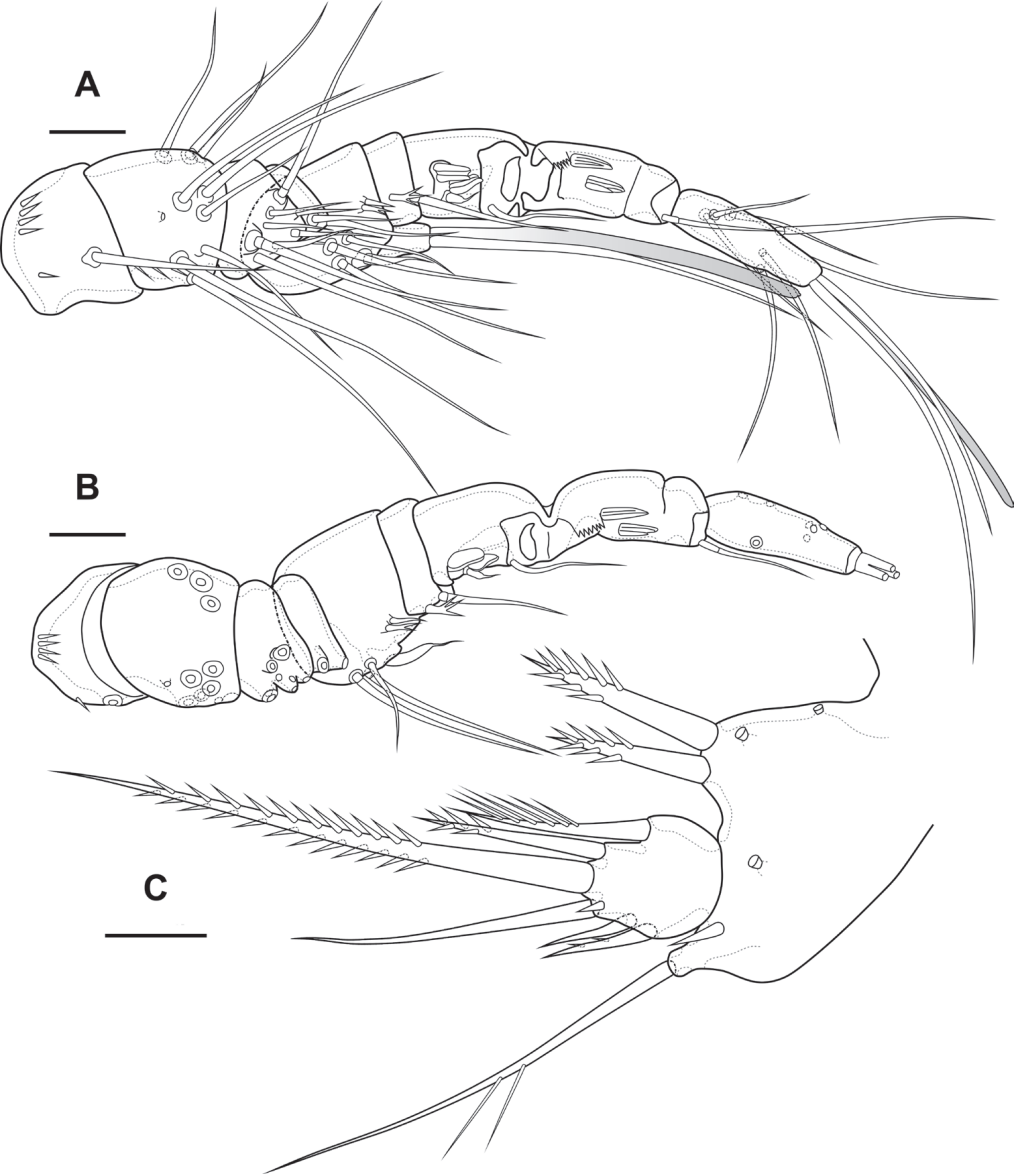
*Bryocamptusabramovae* sp. nov., male **A** antennule, anterior **B** antennule, dorsal **C**P5, anterior. Scale bars: 10 µm.

Antennule (Fig. 17A, B) 10-segmented, haplocer with geniculation between segments 7 and 8. Segments 1, 3, 4, 5, 6, 9, and 10 almost like in *B.minutus*, but more elongated. Segment 2 with small pore on anterior side. Segment 7 with articular plate, with one filiform seta, one small caudate seta and with two modified laminar setae. Segment 8 with proximal short dentate plate and two modified laminar setae. Armature formula: 1-[1],2-[9],3-[8],4-[2],5-[6+(1+ae)],6-[2],7-[2+2 modified],8-[2 modified],9-[1],10-[7+acr].

**Figure 18.**
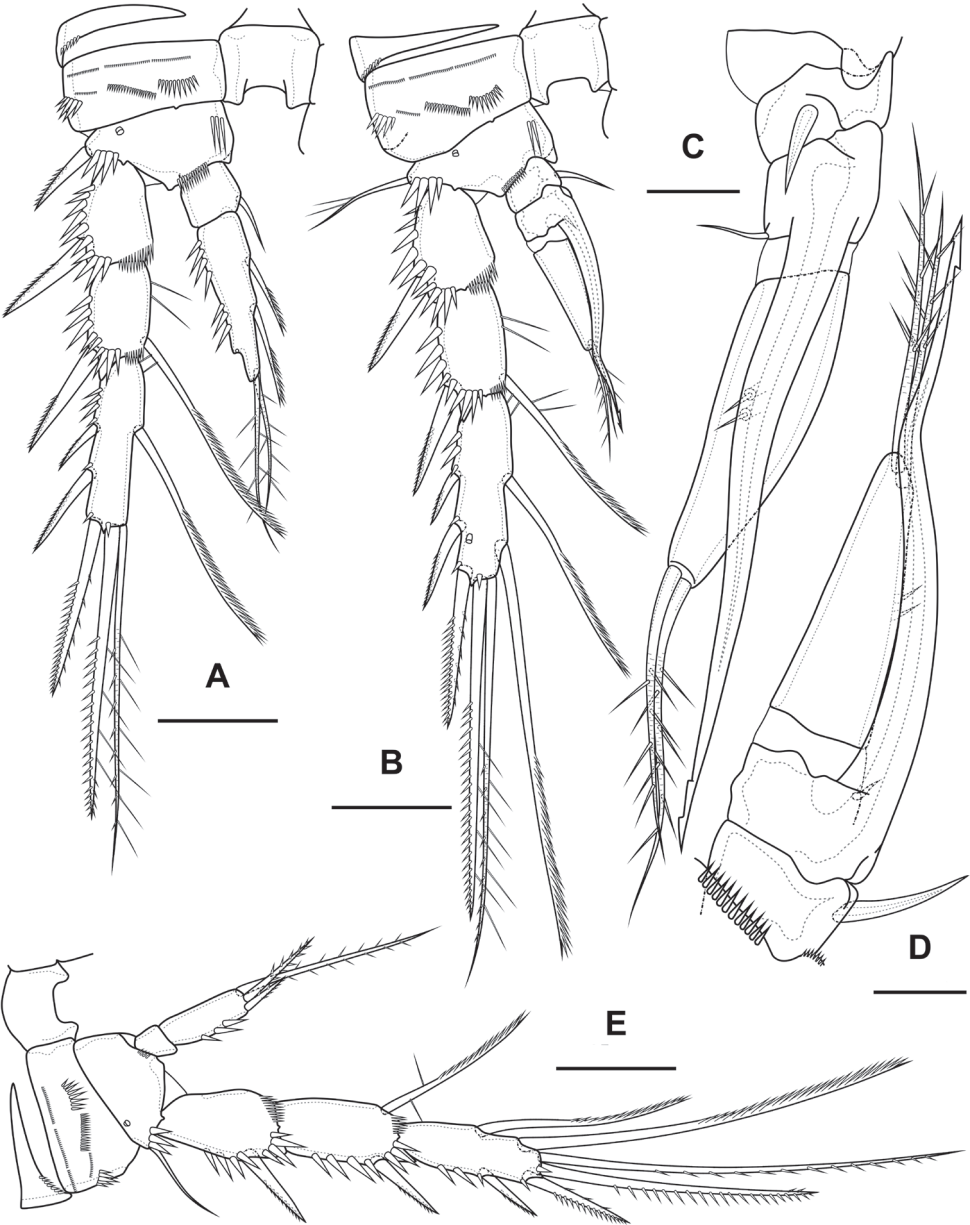
*Bryocamptusabramovae* sp. nov., male **A**P2, anterior **B**P3, anterior **C**P3 endopod, anterior **D**P3 endopod, inner view **E**P4, anterior. Scale bars: 25 µm (**A, B, E**); 10 µm (**C, D**).

P2 (Fig. 18A) as in female, except endopod. Endopod two-segmented. First segment with inner seta. Second segment with notch on distal outer margin, outer spinules, two apical pinnate slender setae and two inner pectinate setae.

P3 (Fig. 18B–D): praecoxa, coxa, intercoxal sclerite as in female. Basis as in female, but with larger inner process. Exopod as in female, but third segment with pore. Endopod three-segmented. First endopodal segment with strong seta. Second endopodal segment with posterior thin seta and long apophysis with double tip. Third segment with two small inner setae and two apical pinnate setae.

P4 (Fig. 18E): praecoxa, coxa, intercoxal sclerite, basis, exopod as in female. Endopod two-segmented; first segment short, unarmed; second segment with outer spinules, spinulose spine, outer apical spiniform spinulose seta and inner apical bipinnate seta.

P5 (Fig. 17C) right and left fused medially. Baseoendopod with three pairs of pores, outer spinule and outer long pinnate seta; endopodal lobe with two strong spinulose apical spines. Exopod with spinule on anterior surface, two equal length outer setae, naked outer subapical seta, long apical spinulose seta, one inner spinulose seta and one long inner pectinate seta with long setules.

##### Variability.

Individuals with two outer spines on the third exopodal segments of P2–P4 were found.

##### Etymology.

This species is named after Ekaterina Abramova, teacher and mentor of the first author.

##### Remarks.

The species is well distinguished from other species of the *B.minutus* group by the presence of only five setae on the endopodal lobe of females P5 and by simple caudal rami with unmodified setae.

#### ﻿ Bryocamptus (Bryocamptus) putoranus sp. nov.

Taxon classificationAnimaliaHarpacticoidaCanthocamptidae

30E8A0B8-0133-5BF5-94D6-46705779EE8E

https://zoobank.org/0591F5CD-A09C-4D37-AC8B-DC1CC93E8B3B

Figs 19 , 20 , 21 , 22 , 23 , 24 , 25 , 26 , 27

##### Material.

***Holotype***: Russia • ♀ dissected on two slides; Russia, Putorana Plateau, large nameless lake in the upper flow of the Neral River; 68.901987°N, 94.170533°E; depth 0.5–1 m; 4 Aug. 2021; E. Chertoprud leg; BP 548/1-a, BP 548/1-b. ***Allotype***: Russia •♂ dissected on one slide; collection data as for holotype; BP 548/2. ***Paratypes***: Russia • ♀ dissected on two slides (BP 548/3-a, BP 548/3-b) and ♂ dissected on one slide (BP 548/4); Putorana Plateau, large nameless lake; 68.898348°N, 94.174442°E; depth 0.5–1 m; 4 Aug. 2021; E. Chertoprud leg.

##### Description.

**Figure 19.**
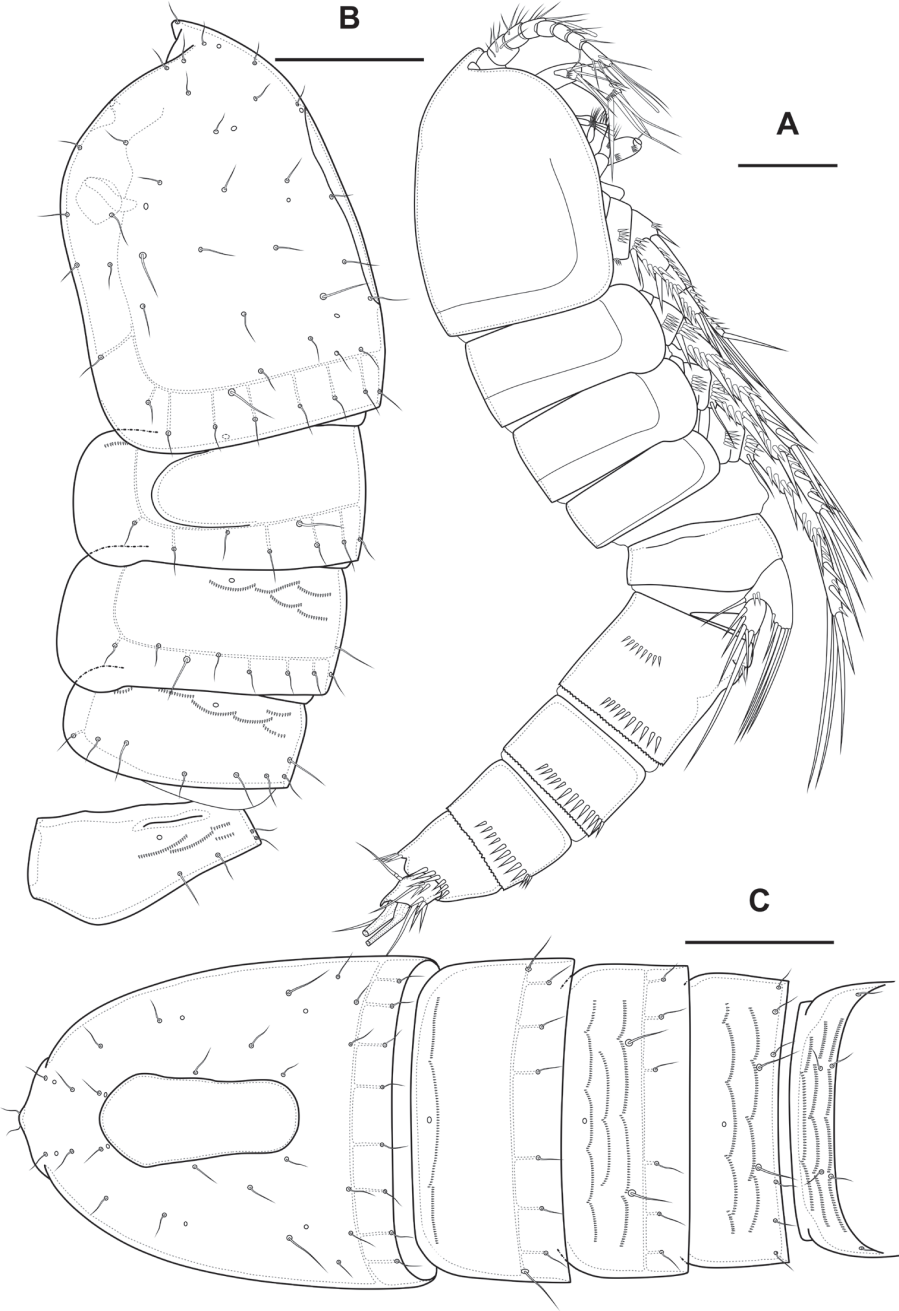
*Bryocamptusputoranus* sp. nov., female **A** habitus, lateral **B** cephalothorax and thoracic somites, lateral **C** cephalothorax and thoracic somites, dorsal. Scale bars: 50 µm.

Female (based on holotype and paratype). Body subcylindrical (Fig. 19A). Total body length from anterior margin of rostrum to posterior margin of caudal rami: 527 µm (*n* = 1). Cephalothorax (Fig. 19B, C; Appendix 1), wider than remaining somites, length 144 µm, largest width 112 µm. Naupliar eye not observed. Rostrum (Fig. 21A) small, fused with cephalothorax, with rounded end, with one pair of sensillae and pore located distal to sensillae. Posterior margin of cephalothorax and all pedigerous somites smooth.

Cephalothorax (Fig. 19B, C; Appendix 1) with dumbbell-shaped dorsal window, seven pairs of pores, seven pairs of sensillae of central group (group C), 13 pairs of sensillae of marginal group (group P) and 21 pairs of ungrouped sensillae (marked as L in Table 4 and in Appendix 1). Second pedigerous somite with lateral windows, dorsal unpaired pore, lateral pair of pores and eight pairs of sensillae. Third pedigerous somite with dorsal unpaired pore, lateral pair of pores and nine pairs of sensillae. Fourth pedigerous somite with dorsal unpaired pore, lateral pair of pores and eight pairs of sensillae. Fifth pedigerous somite with lateral pair of pores and four pairs of sensillae.

**Figure 20.**
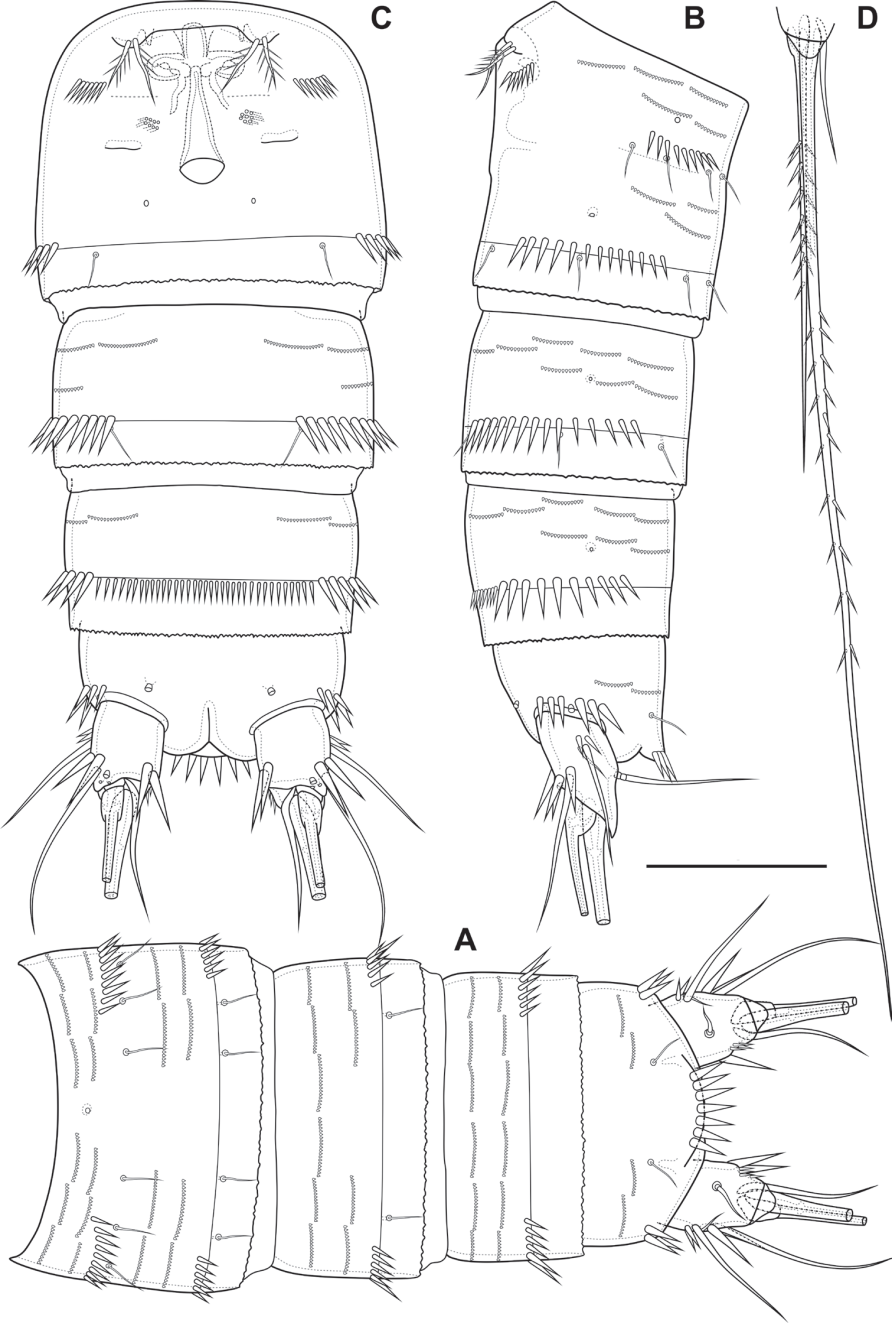
*Bryocamptusputoranus* sp. nov., female **A** abdomen, dorsal **B** abdomen, lateral **C** abdomen, ventral **D** caudal setae, dorsal. Scale bar: 50 µm.

Abdomen (Fig. 20A–C) consisting of genital-double somite, two free abdominal somites and anal somite with caudal rami. All somites except anal somite with wavy posterior margin, on surface with spinular rows. Genital-double somite consists of last thoracic somite and first abdominal somite; wider than long; anterior part with four pairs of sensillae, dorsal unpaired pore, lateral paired pores, ventro-lateral and lateral rows of spinules; posterior part with four pairs of sensillae, pairs of ventral and lateral pores and lateral rows of spinules.

P6 (Fig. 20C) fused with somite with two pinnate setae. Genital field (Fig. 20C) long, laterally with eight-pore sieves; copulatory pore displaced to posterior part of somite, copulatory duct chitinised with two additional tubes, extending proximally to pair of labyrinthic rounded ducts and one chitinised unpaired duct.

Second, third abdominal and anal somites as in *B.minutus*. Anal operculum semilunar, with seven long simple spinules. Caudal rami (Fig. 20A–D). Length/width ratio 1.5, with three ventral pores; with rows of spinules on ventral and dorsal side at base of seta IV and rows spinules at base of setae II and III. Seta I small, located near seta II. Setae IV, V and VI displaced to ventral side of caudal ramus. Apical seta IV (Fig. 20D) bipinnate, with massive bulbous base and “helle Stelle”. Apical seta V long, bipinnate, with “helle Stelle”. Seta VII triarticulated (Fig. 20B).

Antennule (Fig. 20B) similar to that of *Bryocamptusminutus*. Differences expressed in more elongated segments, especially 3^rd^ and 4^th^ segments; one of setae on segment 2 pinnate. Armature formula: 1-[1],2-[9],3-[5],4-[1+(1+ae)],5-[1],6-[3],7-[2],8-[5+acr].

**Figure 21.**
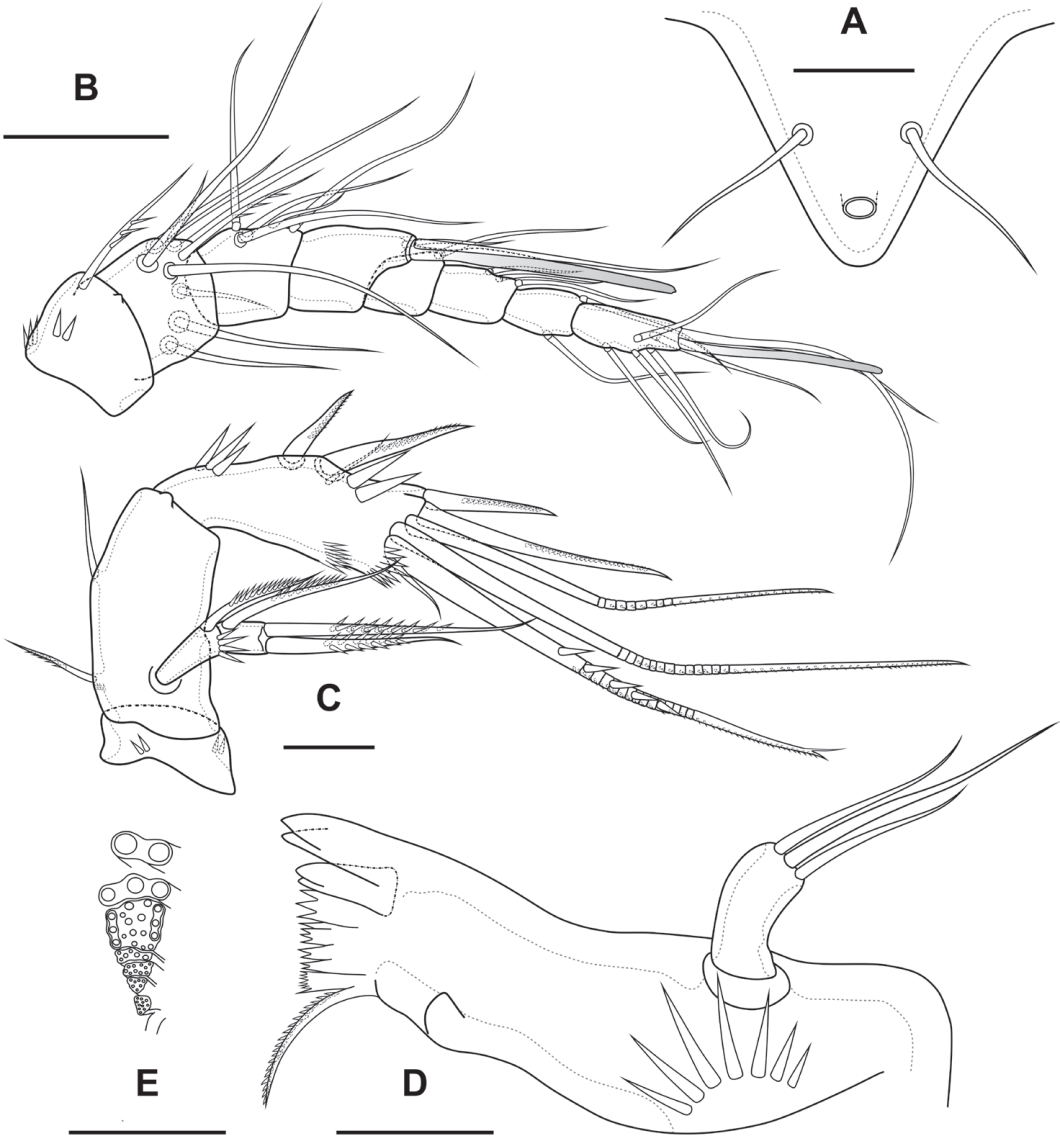
*Bryocamptusputoranus* sp. nov., female **A** rostrum **B** antennule **C** antenna **D** mandible **E** scheme of teeth of mandibular gnathobase. Scale bars: 5 µm (**A**); 10 µm (**B–E**).

Antenna (Fig. 21B) similar to that of *Bryocamptusminutus*. Allobasis and free endopodal segment slightly shorter. Allobasis with proximal outer spinular row, basal seta pinnate; without spinular row at base of endopodal seta.

**Figure 22.**
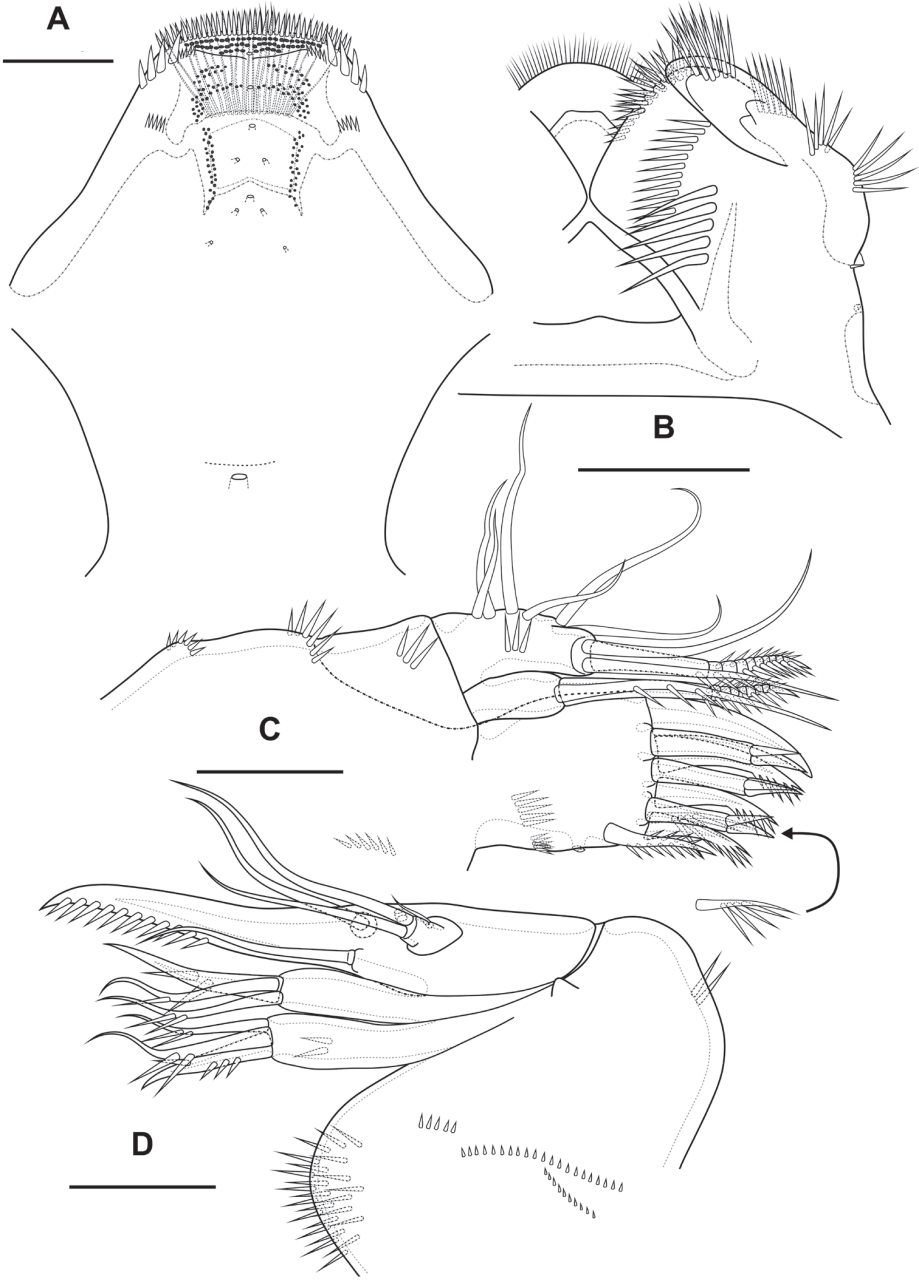
*Bryocamptusputoranus* sp. nov., female **A** labrum, posterior (black dots is bases of spinules) **B** paragnaths, anterior **C** maxillule **D** maxilla. Scale bars: 10 µm.

Labrum (Fig. 22A) similar to that of *Bryocamptusminutus*, but without semicircular spinular row on inner side.

Mandible (Fig. 21D, E). Coxa and gnathobase as in *Bryocamptusminutus*. The palp elongated, with three apical setae.

Paragnaths (Fig. 22B) similar to that of *Bryocamptusminutus*, with only two groups of spinules on anterior side and without proximal spinular row.

Maxillule (Fig. 22C) similar to that of *Bryocamptusminutus*. Coxal endite without spinules; basis with group of spinules.

Maxilla (Fig. 22D) as in *Bryocamptusminutus*, only with slight differences in length and armature of setae.

**Figure 23.**
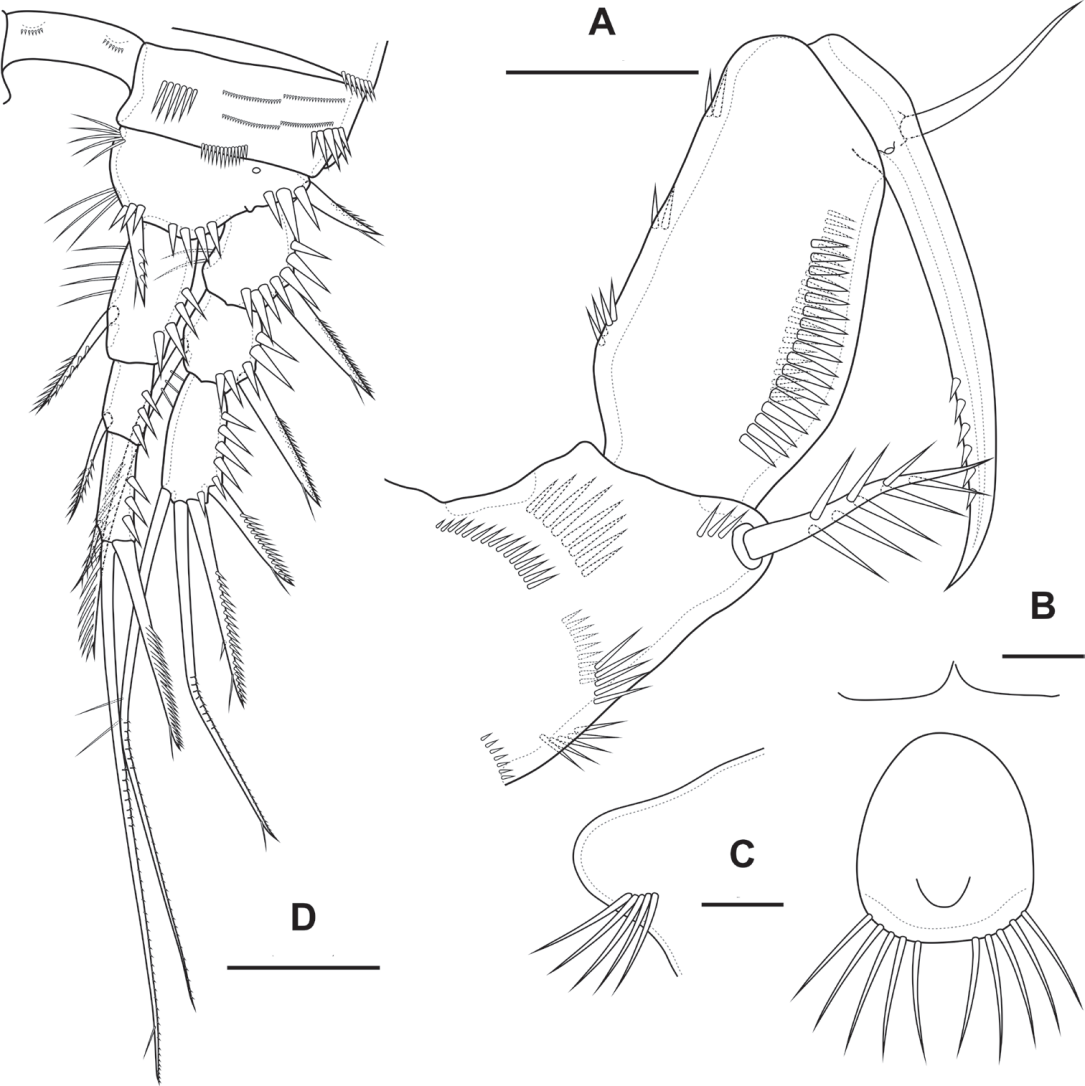
*Bryocamptusputoranus* sp. nov., female **A** maxilliped **B** cuticular process between maxillipeds and P1, ventral **C** cuticular process between maxillipeds and P1, lateral **D**P1. Scale bars: 10 µm (**A**); 5 µm (**B, C**); 25 µm (**D**).

Maxilliped (Fig. 23A) similar to that of *Bryocamptusminutus*. Differences are only in shorter syncoxa and basis.

Cuticular process between maxillipeds and P1 (Fig. 23B, C) extremely high, with long spinules, five spinules on each side. Spinules on posterior margin.

P1 (Fig. 23D) almost like in *Bryocamptusminutus*. Basis with two inner groups of long spinules. First exopodal segment with inner spinules. First endopodal segment reaching end of second exopodal segment. Second endopodal segments with smooth inner side. Differences also noticeable in shorter exopodal and endopodal segments.

**Figure 24.**
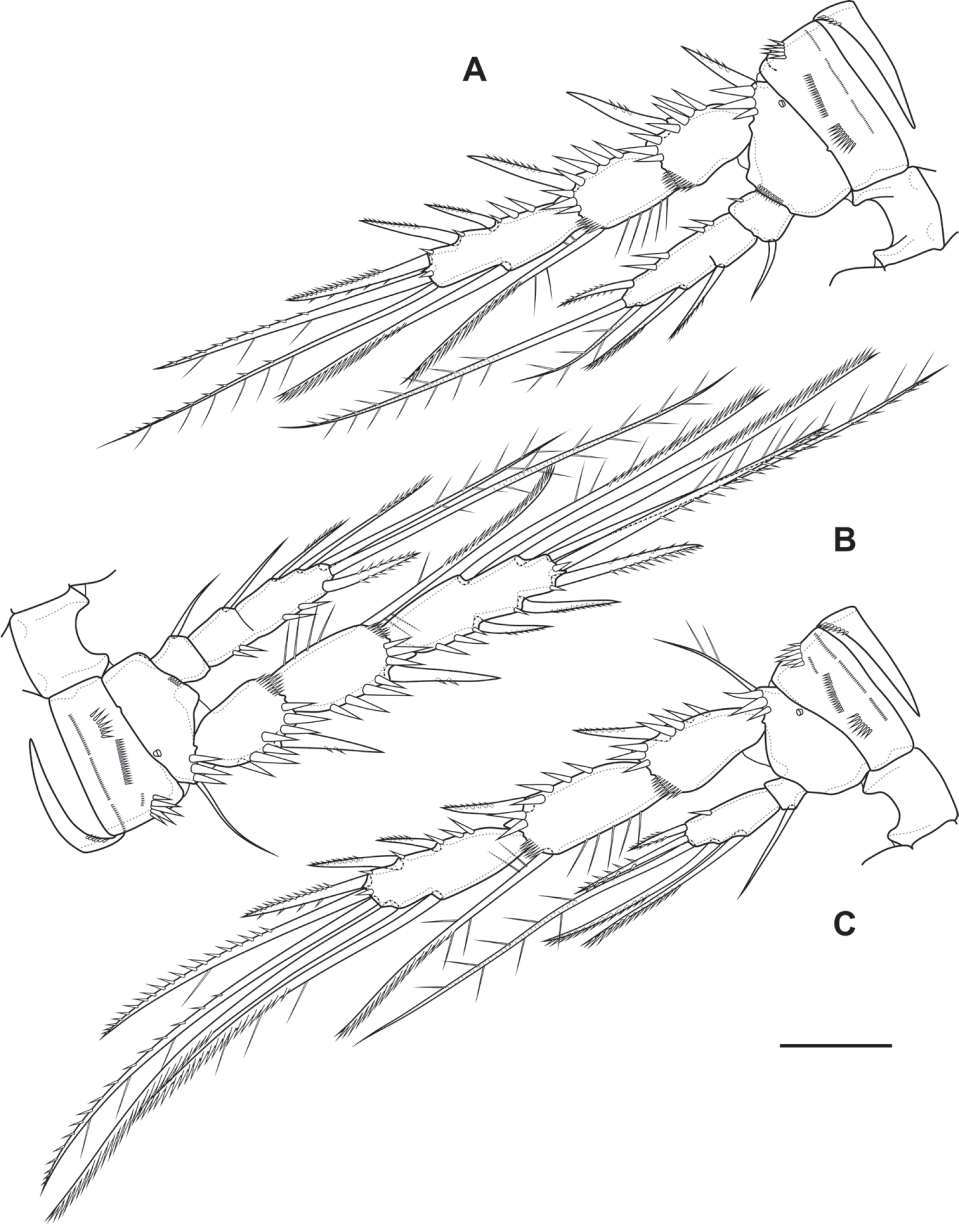
*Bryocamptusputoranus* sp. nov., female **A**P2, anterior **B**P3, anterior **C**P4, anterior. Scale bar: 25 µm.

P2 (Fig. 24A; Table 3). Praecoxa with row of spinules. Coxa with one lateral row of large spinules, two anterior rows of large spinules and four anterior rows of small spinules. Intercoxal sclerite naked. Basis with proximal pore, rows of spinules at base of endopod and exopod; with outer spine. All endopodal and exopodal segments with outer spinules. Exopod three-segmented; first exopodal segment with outer spinulose spine, apically with frill; second segment with outer spinulose spine, inner pectinate seta, inner slender spinules and apical frill; third segment with three outer spinulose spines, two apical setae and one inner pectinate seta. Endopod two-segmented; first segment with inner seta; second segment with distinct border between ancestral segments, outer spinulose spine, two apical pinnate setae and two inner pectinate setae.

**Table 3.** P1 – P4 armature of *Bryocamptusputoranus* sp. nov.

	Female endopod	Male endopod	Exopod
P1	1; 1; 1,1,1	1; 1; 1,1,1	0; 1; 0,2,2
P2	1; 2,2,1	1; 2,2,0	0; 1; 1,2,2-3
P3	1; 3,2,1	1; 1+ ap; 2?,2,0	0; 1; 2,2,3
P4	1; 2,2,1	0; 0,2,1	0; 1; 2,2,2-3

P3 (Fig. 24B; Table 3). Praecoxa with spinular row. Coxa with one lateral row of large spinules, two anterior rows of large spinules and four anterior rows of small spinules. Intercoxal sclerite without spinules. Basis with outer seta, proximal pore, and rows of spinules at base of endopod and exopod. Exopod three-segmented; first exopodal segment with outer spinulose spine, outer spinules, apically with frill; second segment with outer spinulose spine, outer spinules, inner pectinate seta, inner slender spinules and apical frill; third segment with three outer spinulose spines, two apical setae and two inner pectinate setae. Endopod two-segmented; first segment with inner seta; second segment with distinct border between ancestral segments, outer spinules, outer spinulose spine, two apical pinnate setae and three inner setae.

P4 (Fig. 24C; Table 3). Praecoxa with spinular row. Coxa with one lateral row of large spinules, two anterior rows of large spinules and four anterior rows of small spinules. Basis with outer seta, proximal pore, rows of spinules at base of exopod. Exopod three-segmented; first exopodal segment with outer spinulose spine, outer spinules, apically with frill; second segment with outer spinulose spine, outer spinules, inner pectinate seta, inner slender spinules and apical frill; third segment with three outer spinulose spines, two apical setae and two inner pectinate setae. Endopod two-segmented; first segment with inner seta, second segment with outer spinule, outer spinulose spine, apical spiniform spinulose seta, apical pinnate seta and two inner pectinate setae.

**Figure 25.**
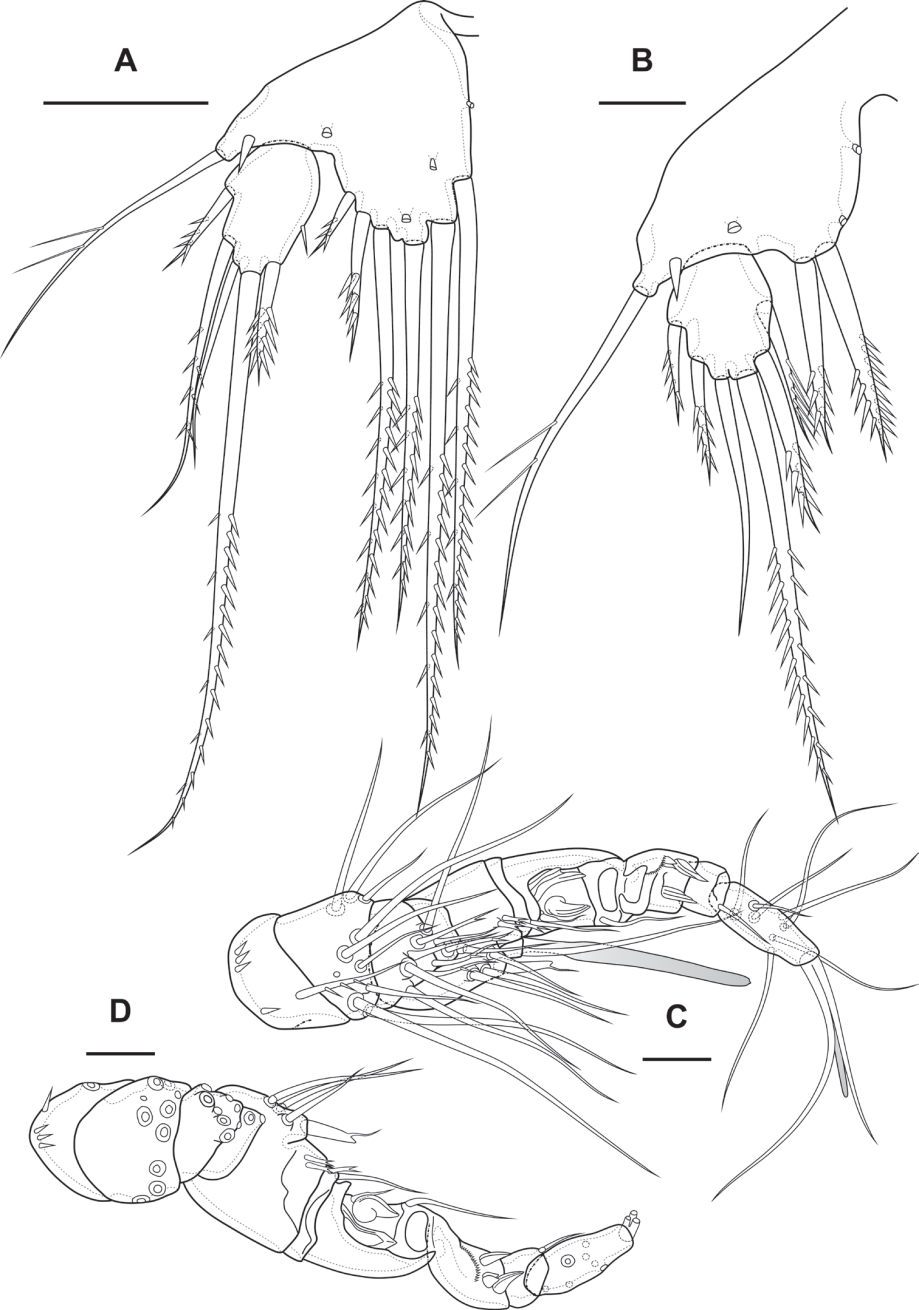
*Bryocamptusputoranus* sp. nov., female **A**P5, anterior; male **B**P5, anterior **C** antennule, anterior **D** antennule, dorsal. Scale bars: 25 µm (**A**); 10 µm (**B–D**).

P5 (Fig. 25A) with separate right and left baseoendopods. Baseoendopod reaching ~ 1/2 of exopodal segment; with four pores, spinule at base of outer seta; outer seta of basis pinnate, long. Endopodal lobe with four long bipinnate setae and two short bipinnate setae V and VI; with small process that may be pore between setae III and IV. Exopod with inner spinule, inner strong pinnate seta, long apical pinnate seta, naked subapical seta and two pinnate outer setae.

**Figure 26.**
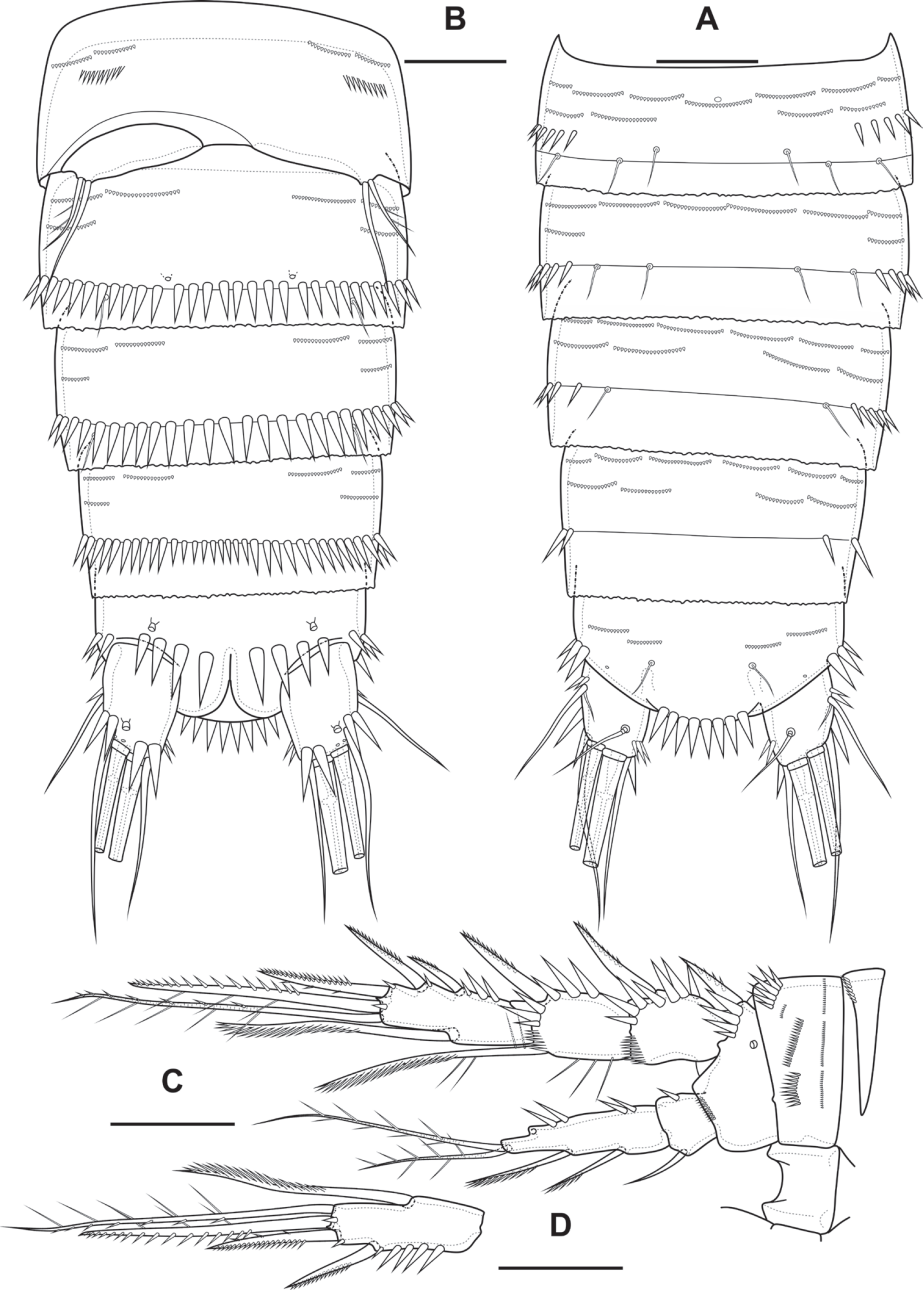
*Bryocamptusputoranus* sp. nov., male **A** abdomen, dorsal **B** abdomen, ventral **C**P2, anterior **D**P2Exp3, variance Scale bars: 25 µm.

Male. Sexual dimorphism expressed in the antennule, P2–P6, genital segmentation and ornamentation, shape of caudal rami. Cephalothorax and thoracic somites as in female. P6 (Fig. 26B) two asymmetric flaps fused to the somite, with one naked and one pinnate setae. Differences from female in abdomen structure as follows (Fig. 26A, B): first abdominal somite free; first to third abdominal somites with spinular row encircling somite ventrally and laterally; anal somite with ventral spinules; caudal rami with normal setae IV and V; anal operculum with eight simple spinules.

Antennule (Fig. 25C, D) 10-segmented, haplocer with geniculation between segments 7 and 8. Segments 1, 3, 4, 5, 6, 9, and 10 similar to those of *B.minutus*, but differ in length. Segment 2 with small pore on anterior side. Segment 7 with articular plate, with one filiform seta, one small caudate seta and with two large modified laminar setae. Segment 8 with proximal long dentate plate and three modified laminar setae. Armature formula: 1-[1],2-[9],3-[8],4-[2],5-[6+(1+ae)],6-[2],7-[2+2 modified],8-[3 modified],9-[1],10-[7+acr].

P2 (Fig. 26C, D) as in female, except endopod. Endopod two-segmented. First segment with outer spinule and inner seta. Second segment with notch on distal outer margin, outer spinules, two apical pinnate slender setae and two inner pectinate setae.

**Figure 27.**
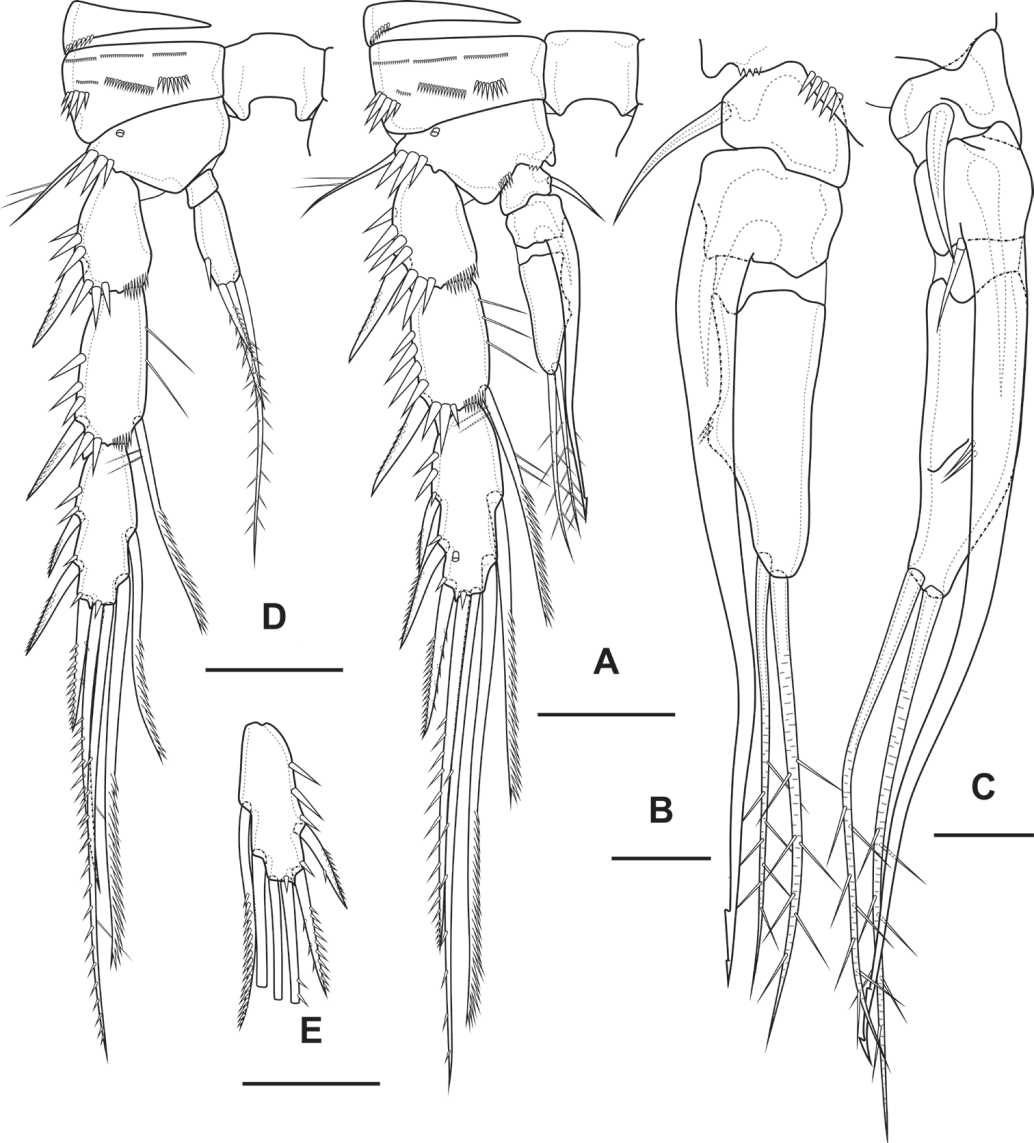
*Bryocamptusputoranus* sp. nov., male **A**P3, anterior **B**P3 endopod, anterior **C**P3 endopod, inner view **D**P4, anterior **E**P4Exp3, variance. Scale bars: 25 µm (**A, D, E**); 10 µm (**B, C**).

P3 (Fig. 27A–C): praecoxa, coxa, intercoxal sclerite as in female. Basis as in female, but with inner process. Exopod as in female, but third segment with pore. Endopod three-segmented. First endopodal segment with strong seta. Second endopodal segment with posterior seta and long apophysis with double tip. Third segment with probably two small inner setae and two apical pinnate setae.

P4 (Fig. 27D, E): praecoxa, coxa, intercoxal sclerite, basis, exopod as in female. Endopod two-segmented; first segment short, unarmed; second segment with outer spinule, spinulose spine, outer apical spiniform spinulose seta and inner apical bipinnate seta.

P5 (Fig. 27B) right and left fused medially. Baseoendopod with three pairs of pores, outer spinule and outer long pinnate seta; endopodal lobe with two strong spinulose apical spines. Exopod with two outer spinulose setae, naked outer subapical seta, long apical spinulose seta, one inner spinulose seta and one inner pectinate seta with long setulles.

##### Variability.

Individuals with two outer spines on the third exopodal segment of P2 and P4 were found (Figs 26D, 27E). One female was also found with both simple and bifid spinules on the anal operculum.

##### Etymology.

The species is named so because it was found on the Putorana Plateau.

##### Remarks.

The species as a whole is similar to *B.hutchinsoni*, including the structure of caudal rami; however, it differs well in two-segmented endopods P2 and P3. Another find of *B.hutchinsoni* (Carter 1944) differs markedly in the structure of its caudal rami and is not similar to *B.putoranus* sp. nov.

## ﻿Discussion

### ﻿*Bryocamptusminutus* species group

We agree with Lang (1948) that the *B.minutus* group can reliably differ from other *Bryocamptus* s. str. species precisely in the structure of the mandibular palp. In addition, it is also necessary to consider the structure of the caudal rami and the anal somite. Species of this group always have a small number (5–15) of large spinules on the anal operculum, and often these spinules are bifid, as if two spinules are fused together. Bifid spinules can also be a characteristic feature of some species (*B.minutus*, *B.aberrans* Apostolov & Pesce, 1991, *B.abramovae* sp. nov.) (Apostolov and Pesce 1991), and are also often found in species with simple spinules as a result of intraspecific variability (*B.hutchinsoni*, *B.putoranus* sp. nov., *B.vejdovskyiminiformis* Kiefer, 1934) (Kiefer 1934). The anal somite of females of this species lacks ventral spinular rows.

At the same time, the use of armature and segmentation of swimming legs is rather doubtful. In species of this group, there is often variability in the number of spines on the distal exopodal segments P2–P4, especially P4 (*B.minutus*, *B.putoranus* sp. nov., *B.abramovae* sp. nov.). The three-segmented endopods of the swimming legs are also partially or completely fused in some species (*B.putoranus* sp. nov., *B.aberrans*) (Apostolov and Pesce 1991).

Based on the structure of the mandibular palp, the shape of P5, the armature of the abdominal somites, the shape of the caudal rami and the armature of the anal operculum, we believe that the *B.minutus* group should include the following species: *B.abramovae* sp. nov., *B.aberrans*, *B.hutchinsoni*, *B.minutus*, *B.pilosus* Flössner, 1989, *B.putoranus* sp. nov., *B.vejdovskyi*. Some species with incomplete descriptions can also most likely be attributed to this group: *B.intercalaris* Shen & Tai, 1973, *B.nenggaoensis* Young, 2010. In particular, descriptions and figures of mandibles are not given for these species; however, according to other characters, they could belong to the group (Shen and Tai 1973, Young 2010). For the species *B.bogoriensis* Kiefer, 1933, *B.borutzkyi* Petkovski, 1969 and *B.washingtonensis* Wilson, 1958, the descriptions are incomplete, so it is difficult to assign them to any group.

Another very similar species is B. (B.) campaneri (Reid, 1994) from Brazil, described only on the female. It resembles representatives of the group in the structure of caudal rami with reduced seta IV and anal somite of female without ventral group of spinules. However, this species has a two-segmented mandibular palp with a seta on the proximal segment (Reid 1994). It is likely that with the discovery and study of males of this species, it will also need to be included in the *B.minutus* species group with an expansion of the group characters.

*Bryocamptusminutus* species group appears to have a Holarctic distribution. In general, among freshwater Harpacticoida, this distribution is characteristic of many genera and groups of species, such as *Canthocamptus* Westwood, 1836 (Novikov and Sharafutdinova 2022), *Pesceus* Özdikmen, 2008, Attheyella (Neomrazekiella) Özdikmen & Pesce, 2006 (Borutzky 1952). The only species outside the Holarctic is *B.nenggaoensis* described from Taiwan (Young 2010). Difficulties arise when considering species with a wide range. Thus, the taxonomic status of many North American forms of species described in the Palearctic, in particular *B.vejdovskyi* and *B.minutus*, is unknown. Wilson mentions this as a problem with *B.minutus-hutchinsoni-vejdovskyi* and points out that there are probably significantly more species. (1956). A step towards solving this problem was the description of *B.pilosus*, related to *B.vejdovskyi* (Flössner 1989), but it is still far from being solved. It is likely that *B.vejdovskyiminiformis* with bifid spinules (Kiefer 1934) is also a separate Nearctic species. Some species from Europe also are described in a large number of varieties and forms (Thallwitz 1916; Lang 1957). Many taxonomists considered these forms and subspecies only intraspecific variability (Lang 1948; Borutzky 1952); however, it may well turn out that they will also be separate species.

Unfortunately, even now, descriptions of freshwater species of Copepoda are very incomplete and rather approximate. Even such significant structures as the antennules of females often are drawn with an incomplete number of setae. Antennules of males are often either not drawn or drawn very superficially. The problem of poor-quality descriptions was discussed by Hamond (1987); when compared with the best descriptions of that time, he wrote: “Subsequent students of freshwater copepods should emulate these authors as far as is technically possible. If they cannot produce drawings as good as theirs they should stay away from the formidably exacting demands of modern taxonomic practice” (Hamond 1987).

We hope that this work can be used in the future to unravel such a complex genus as *Bryocamptus*, and that the authors of original descriptions will not neglect even small, but taxonomically important, details.

### ﻿Analysis of differences between studied species

The conclusions of this chapter are made on the basis of representatives of one population of each species. These characters are fairly stable within the studied populations; however, we cannot say how stable they are over a larger geographical area.

There are very large differences in the ornamentation of the limbs, which is undoubtedly homologous and can be used in taxonomy. However, this should be done with caution, until it is fully understood to what extent these characters are subject to intraspecific variability. Although for other groups of copepods, some elements of micro-ornament have been shown to be very effective in distinguishing closely related species. For example, in the taxonomy of Cyclopidae, ornamentation of antenna allobasis (Fiers and Van de Velde 1984), maxilla basis (Hołyńska et al. 2021), coxa of P4 (Van de Velde 1984) are used widely. Another difficult feature is that during the preparation of specimens or during the life of these crustaceans, some of the spines, especially long ones, can break off, and some wear out, so it is necessary to study at least a few individuals of each species.

There can be two mechanisms for the reduction of groups of spinules. The first is a decrease in the number of spinules until their complete disappearance. This is typical condition for one of the groups of spinules on the first segment of the female antennule, in the studied *Bryocamptus* it is one-two spinules, and for example in *Maraenobiotus* they are already completely absent (Novikov and Sharafutdinova 2020). The second mechanism is a strong decrease in these spines, also up to complete disappearance. Here, the best example is the ornamentation of the coxae of P2–P4. Several rows of small spinules are clearly visible in primitive Canthocamptidae, as *Canthocamptus* or *Attheyella* (Novikov and Sharafutdinova 2022), may be almost invisible in *B.minutus* group, and completely absent, for example, on the coxa of P4 of B. (Rheocamptus) pygmaeus (Sars, 1863) (Novikov and Fefilova 2021).

The ornamentation of the cephalothorax and thoracic somites showed significant differences between the three species studied, shown in Table 4.

**Table 4.** Table of differences in the composition of pores and sensillae on cephalothorax and thoracic somites (designations in Appendix 1).

Somite	Cephalothorax																
Species	I	VI	XI	XIV	P 1	P 3	P 10	P 13	P 17	L 6	L 9	L 16	L 18	L 19	L 29	L 35	L 36
*B.minutus*	-	+	+	+	+	+	+	+	+	+	+	+	+	-	+	+	+
*B.abramovae*	+	-	-	-	-	-	-	-	-	-	-	-	-	-	-	-	-
*B.putoranus*	+	-	-	-	+	+	+	+	+	+	+	+	+	+	+	+	+
**Somite**	**PS2**		**PS3**						**PS4**						**PS5**		
Species	3	8	II	2	7		10		II	4		8	9		II	1	
*B.minutus*	+	+	+	+	+		-		+	+		+	+		+	+	
*B.abramovae*	-	-	-	-	-		-		-	-		-	-		-	-	
*B.putoranus*	+	+	+	+	+		+		+	+		+	+		+	+	

The demonstrated interspecific variability opens up great scope for the separation of complex groups of species. However, the high variability in the structure of the integument complex (composition of sensillae and pores on somites) between closely related species impairs its applicability in phylogenetic reconstructions. *Bryocamptusabramovae* sp. nov. has a greatly reduced number of these elements, despite the absence of other major differences from the other two species. *Bryocamptusputoranus* sp. nov. and *B.minutus* have an almost identical composition of sensillae and pores on somites. It is also possible for some taxa of copepods that pores (but not sensillae) on somites may appear *de novo* within some lineages, for example, in the family Artotrogidae Brady, 1880 (Siphonostomatoida), species of which have a huge number of large pores on somites (McKinnon 1988).

The rostrum also has significant interspecific variability. The studied species differ in the presence/absence of the pore, its position, and the shape of the distal margin.

Antennules of females have predominantly morphometric differences in the shape of the segments and the length of the setae. Also, one of the setae on the second segment in *B.abramovae* sp. nov. and *B.putoranus* sp. nov. is armed with spinules, in contrast to *B.minutus*.

The antenna also differs significantly in the shape of the segments. The most variable part is the allobasis. Depending on the species, the presence/absence of groups of spinules at the bases of the setae, as well as the armature of the proximal seta of the allobasis, varies. The labrum is almost the same in the studied species, except for a semicircular row of spines on the posterior surface of *B.minutus*.

Mandibles have long been considered one of the most important elements in harpacticoid taxonomy (Lang 1948). In the studied species, differences were found in the number of apical setae on the mandibular palp and in the presence of a group of spinules on the palp, which are absent only in *B.putoranus* sp. nov. Interestingly enough, the studied species have an absolutely identical structure of gnathobases down to the number of small spinules of dental batteries, which probably indicates an identical type of diet. Here it is important to take into account that the gnatobases are quite strongly obliterated over time, which was found in some individuals of *B.minutus*. Therefore, to study them, relatively recently molted individuals are needed. Paragnaths in the studied species differ in shape and number of outer and anterior rows of spinules.

Three groups of spinules are subject to interspecific variability on maxillules, one of which is on the coxal endite, and the other two are on the basis. As with mandibles, some setae of the arthrite are also subject to wear. Therefore, characteristic strong setae with a pectinate end cannot be found in a number of individuals of the same species (Fig. 3B). It should also be noted that in our previous works we have always missed one of the setae of arthrite, which bears very long spinules. Re-examination of the material showed that this seta is present in all species previously described by us: *Maraenobiotussupermario* Novikov & Sharafutdinova, 2020, *Mesopsyllusglacialis* Novikov & Sharafutdinova, 2021, *Heteropsyllusspiridonovi* Novikov & Sharafutdinova, 2021 and *Heteropsyllusspongiophilus* Novikov & Sharafutdinova, 2021.

Maxilla and maxilliped turned out to be identical in ornamentation, differing only in different shape of segments, length of setae, and, to some extent, armament of setae. The processes between the maxillipeds and P1 differ in shape, height, and number of spinules. Thus, *B.minutus* has the largest number of spines that extend onto the anterior side of the process. *Bryocamptusputoranus* is notable for its unusually high process.

P1 is quite different in the studied species. In addition to differences in the shape and length of the segments, the species also differ in the presence/absence of two inner and one frontal groups of spinules on the basis. The inner surface of the exopod and endopod is also armed to varying degrees in different species.

P2–P4 of females, in addition to segmentation, the shape of the segments, and the number of outer spines on the third segment of the P4 exopod, also differ in microcharacters. Intercoxal sclerite of P2 of *B.minutus* has two large spinules. Coxae P2–P4 of *B.abramovae* sp. nov. and *B.putoranus* sp. nov. have an additional group of large spinules. The P2–P3 basis of *B.abramovae* sp. nov. has an inner group of long spinules and a relatively large inner process. The basis of P4 of *B.putoranus* sp. nov. lacks a row of spinules near the base of the endopod. The outer spines of P2–P3Exp1-Exp2 of *B.minutus* are naked, unlike the other two species. P2–P4Exp3 of *B.minutus* have a pore. P2 and P4 of males have approximately the same differences as in females. Only the P4Enp2 of *B.minutus* is distinguished by the presence of four setae, instead of three in *B.abramovae* sp. nov. and *B.putoranus* sp. nov.

The structure of the P3 endopod, on closer examination, can be one of the most important taxonomic characters distinguishing closely related species. In particular, for the genus *Lourinia* Wilson, 1924, closely related to Canthocamptidae, a very strong interspecific variability in the P3 apophysis was described recently; it can vary in length and curvature, as well as in the shape of the tip (Karaytuğ et al. 2021). The three studied species also have significant differences in the structure of the endopod. They differ considerably in elongation, *B.putoranus* sp. nov. and *B.abramovae* sp. nov. have relatively shortened segments. *Bryocamptusputoranus* sp. nov., in addition to this, has a large outgrowth on the third segment, while in the other two species the inner edge of the segment is even. *Bryocamptusminutus* has a pore on the third segment. The shape and length of the apophysis also varies considerably. The absolute length of the apophysis in lateral view and the ratio to the length of the third endopodal segment, respectively: *B.minutus* 77 µm and 2.02; *B.abramovae* sp. nov. 56 µm and 1.80; *B.putoranus* sp. nov. 70 µm and 2.59.

P5 of females of the studied species also differ significantly. First of all, the shape of the endopodal lobe and exopod and the length of the setae. *Bryocamptusabramovae* sp. nov. lacks the inner seta of the endopodal lobe. The exopod of *B.minutus* bears several spinules on the anterior surface. P5 of males are very similar and differ in the shape of the exopods and the armature of the exopodal setae.

P6 of females almost do not differ. However, the P6 of males of *B.putoranus* sp. nov. bears only two setae instead of three. The genital field of females of different species differs primarily in proportions. Abdominal somites of *B.abramovae* sp. nov. has a reduced number of sensillae, as is the case with thoracic somites. The armature of the anal operculum also varies: in *B.minutus* with long bifid spinules, in *B.abramovae* sp. nov. with short bifid spinules, and in *B.putoranus* sp. nov. with long simple spinules.

### ﻿Relationships between caudal rami of females and antennules of males

One of the most interesting details found is the very close relationship between the shape of the caudal rami and the shape of the male antennules. During mating, the antennules of males of some harpacticoids, in particular most canthocamptids, are used to grasp the caudal setae of females (Wolf 1905). To this end, many segments of the male antennule are strongly modified. A joint is formed between segments 7 and 8, and the segments themselves in Canthocamptidae bear modified laminar setae, probably necessary to increase the efficiency of capturing the female. The large segment 5 probably serves more as a location for the large muscles brought directly to the joint. The least modified antennules among Canthocamptidae can be found in the genus *Canthocamptus*, where all laminar setae have a standard appearance, and the shape of the caudal rami of females does not undergo any modification (Novikov and Sharafutdinova 2022).

Of the studied species, females of *B.abramovae* sp. nov. have the least modified caudal rami. This finds a close relationship with male antennules, which have simple segments 7 and 8, as well as unmodified laminar setae on these segments. Females of B.*putoranus* sp. nov. have caudal setae displaced to the ventral side. This is reflected in a slightly altered shape of segments 7 and 8 of the male antennule, as well as in a noticeable increase in laminar setae on segment 7. *Bryocamptusminutus* has the most interesting structure of these parts. Females have strongly displaced apical setae, while male on segment 8 has two strongly enlarged laminar setae, one of which forms a kind of elongated plate, which is probably necessary for close grasping of displaced apical setae from below.

The similar shape of the caudal rami of *B.minutus* and *B.putoranus* sp. nov. could suggest that this character is a synapomorphy of these species. However, the mechanisms that allow males to copulate more effectively with a female are completely different. In *B.minutus*, development reaches laminar setae on segment 8, while in *B.putoranus* sp. nov., on segment 7. Probably, the mating efficiency strongly depends on the coevolution of these two parts; different mechanisms for increasing this efficiency most likely indicate the convergent acquisition of displaced apical caudal setae. This also emphasizes the importance of the detailed illustration of male antennules in species descriptions.

However, the question arises, why should females acquire caudal branches that are difficult to grasp? This is an example of an evolutionary sexual arms race between the sexes of the same species, also noted for members of *Maraenobiotus* (Brancelj and Karanovic 2015). The reasons for such evolutionary mechanisms are not yet fully understood. A fairly well-studied example is the sexual arms race in water striders (Arnqvist and Rowe 2002a; Perry et al. 2017). Male water striders can keep females for quite a long time, impairing their survival (impairs the efficiency of foraging and defense against predators) (Rowe et al. 1994). For prolonged mating, males have modified genitals and abdomen (Arnqvist and Rowe 2002b).

As with water striders, it is probably beneficial for the *Bryocamptus* male to keep the female as long as possible to protect the female from fertilization by other males. At the same time, this is not beneficial for the female, since it most likely has a negative effect on protection from predators and the efficiency of foraging. Accordingly, females acquire such caudal rami that males cannot hold them for a long time. And males acquire modified antennules in parallel.

The incompatible shape of the caudal branches of the females and the antennules of the males serve as a mechanism for reproductive isolation (premating isolation). This is one of the microevolutionary processes leading to rapid allopatric and sympatric speciation, for example, in the extremely diverse Baikalian Moraria (Baikalomoraria) Borutzky, 1931 (Borutzky 1952). Therefore, the different shape of the caudal rami and their setae within the same species most likely indicates the presence of already divergent species, which has already been described for *Maraenobiotus* (Brancelj and Karanovic 2015). But it is probably much more common, for example, forms with different caudal rami are described in Attheyella (Attheyella) tahoensis Bang, Baguley & Moon, 2015 (Bang et al. 2015), and in different species of *Kikuchicamptus* Novikov & Sharafutdinova, 2022 (Chang and Ishida 2001).

## Supplementary Material

XML Treatment for Subgenus Bryocamptus

XML Treatment for Bryocamptus (Bryocamptus) minutus

XML Treatment for Bryocamptus (Bryocamptus) minutusminutus

XML Treatment for Bryocamptus (Bryocamptus) abramovae

XML Treatment for Bryocamptus (Bryocamptus) putoranus

## Figures and Tables

**Figure A1.**
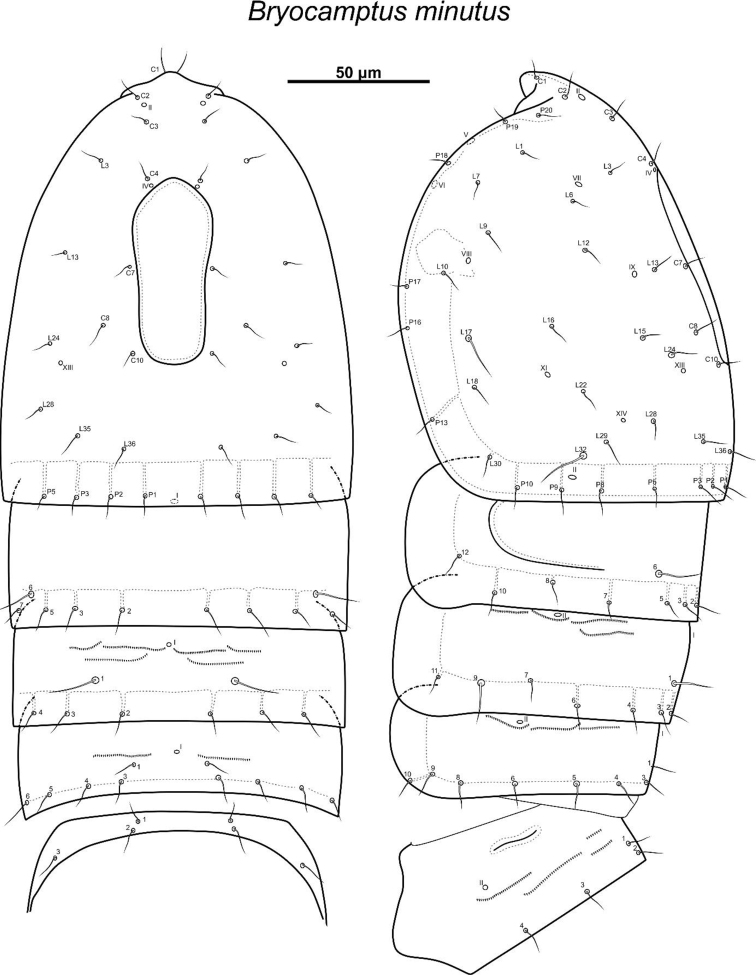
*Bryocamptusminutus*.

**Figure A2.**
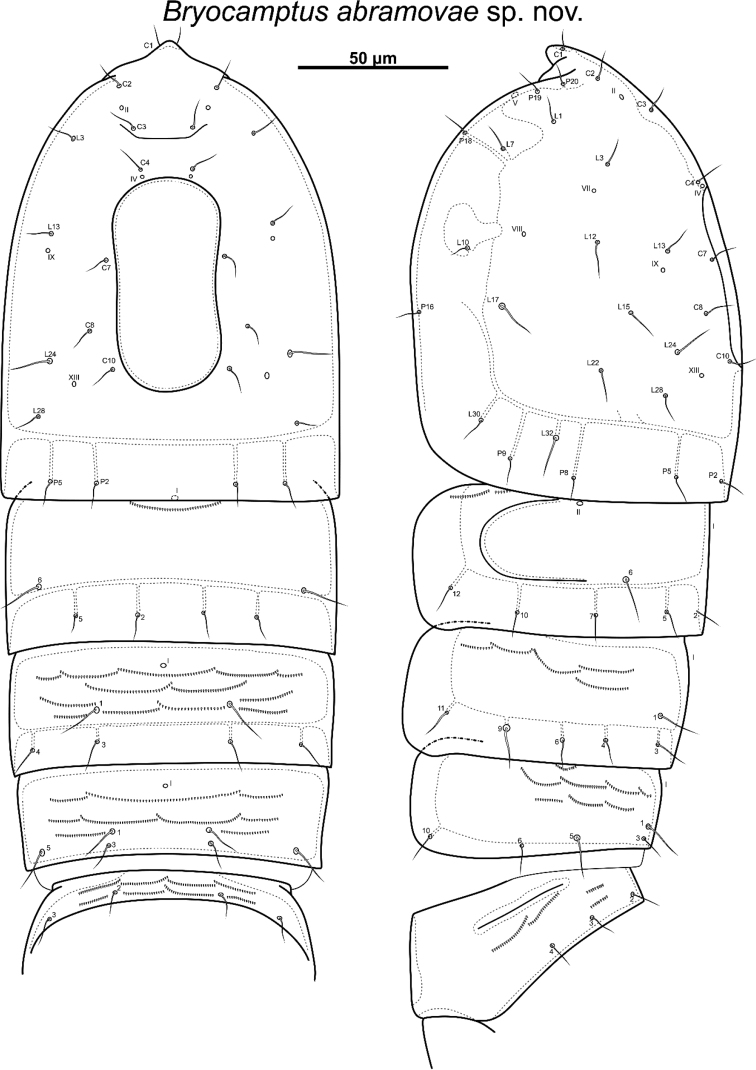
*Bryocamptusabramovae* sp. nov.

**Figure A3.**
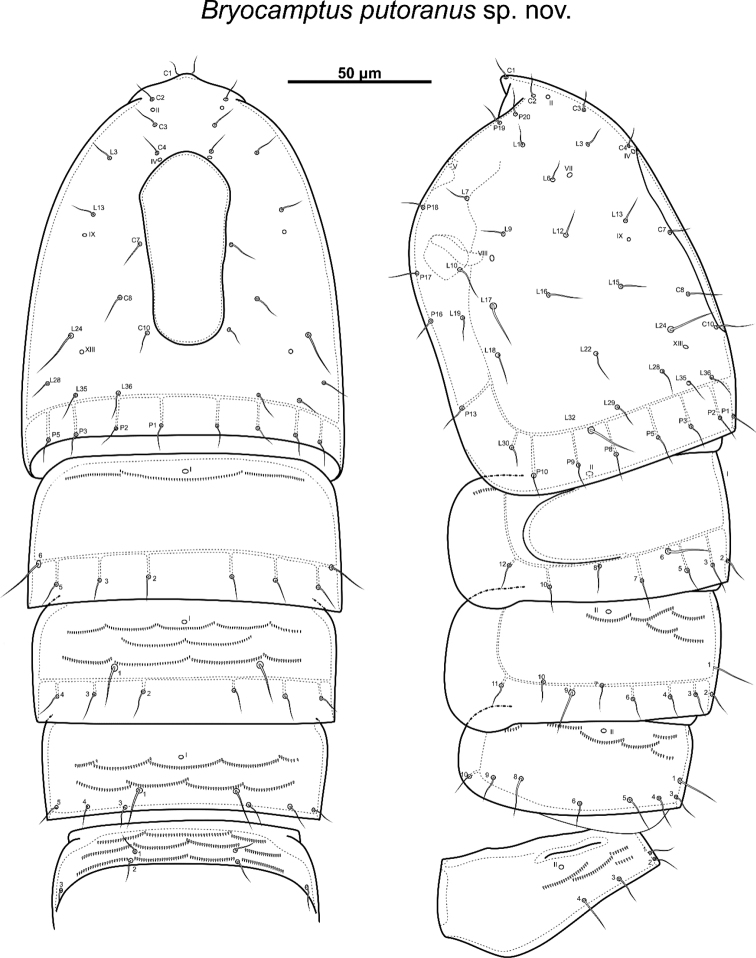
*Bryocamptusputoranus* sp. nov.

## ﻿Appendix 1

Numbering of integumental sensillae and pores of cephalothorax and thoracic somites of the studied species.

The numbering of pores and sensillae on somites is original and based on the structure of the integument of several freshwater species of Canthocamptidae. Roman numerals (for pores) or Arabic numerals (for sensillae) are used for numbering integumental elements. The designations for cephalothorax sensillae C, P, and L are used to simplify homology. Group P is the sensillae adjacent to the edge of the cephalothorax. Group C is the sensillae, which are located near the medial axis and the dorsal window. The notation L is used for all other sensillae.
